# Ciliary IFT80 balances canonical versus non-canonical hedgehog signalling for osteoblast differentiation

**DOI:** 10.1038/ncomms11024

**Published:** 2016-03-21

**Authors:** Xue Yuan, Jay Cao, Xiaoning He, Rosa Serra, Jun Qu, Xu Cao, Shuying Yang

**Affiliations:** 1 Department of Oral Biology, School of Dental Medicine, University of Buffalo, The State University of New York, 3435 Main Street, Buffalo, New York 14214, USA; 2 USDA Grand Forks Human Nutrition Research Center, Grand Forks, North Dakota 58202, USA; 3 Department of Cell, Developmental, and Integrative Biology, University of Alabama at Birmingham, Birmingham, Alabama 35294, USA; 4 Department of Pharmaceutical Sciences, University at Buffalo, The State University of New York, New York 14214, USA; 5 Department of Orthopaedic Surgery, Johns Hopkins University School of Medicine, Baltimore, Maryland 21205, USA; 6 Developmental Genomics Group, New York State Center of Excellence in Bioinformatics and Life Sciences, University of Buffalo, The State University of New York, New York 14203, USA

## Abstract

Intraflagellar transport proteins (IFT) are required for hedgehog (Hh) signalling transduction that is essential for bone development, however, how IFT proteins regulate Hh signalling in osteoblasts (OBs) remains unclear. Here we show that deletion of ciliary *IFT80* in OB precursor cells (OPC) in mice results in growth retardation and markedly decreased bone mass with impaired OB differentiation. Loss of IFT80 blocks canonical Hh–Gli signalling via disrupting Smo ciliary localization, but elevates non-canonical Hh–Gαi–RhoA–stress fibre signalling by increasing Smo and Gαi binding. Inhibition of RhoA and ROCK activity partially restores osteogenic differentiation of *IFT80*-deficient OPCs by inhibiting non-canonical Hh–RhoA–Cofilin/MLC2 signalling. Cytochalasin D, an actin destabilizer, dramatically restores OB differentiation of *IFT80*-deficient OPCs by disrupting actin stress fibres and promoting cilia formation and Hh–Gli signalling. These findings reveal that IFT80 is required for OB differentiation by balancing between canonical Hh–Gli and non-canonical Hh–Gαi–RhoA pathways and highlight IFT80 as a therapeutic target for craniofacial and skeletal abnormalities.

## Abstract (web summary)

Primary cilia are highly conserved microtubule-based organelles that play essential roles in several cellular processes including osteogenesis. Here the authors show that intraflagellar protein IFT80 regulates osteoblast differentiation by balancing signalling though the canonical and non-canonical Hedgehog pathways.

## Graphical abstract

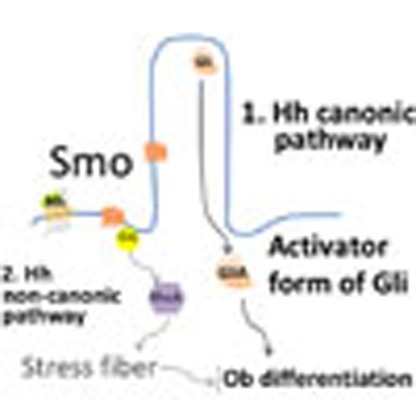

Primary cilia are highly conserved microtubule-based organelles that project from the cell surface into the extracellular environment and play essential roles in vertebrate development, cellular differentiation, proliferation, sensory transduction and homeostasis^1^,^2^. Cilia formation and function requires effective intraflagellar transport (IFT), a bidirectional driving system run by IFT protein complexes and two kinds of motors (kinesin and dynein)^3^. Primary cilia and IFT proteins are present on almost all vertebrate cells including osteoblasts (OBs)^4^. Growing evidence has shown that primary cilia and IFT proteins are integral to the aetiology of skeletal and craniofacial disorders, owing to their role in bone related signal transduction, such as hedgehog (Hh) signalling^5^. Other studies demonstrated that IFT proteins are also involved in the regulation of bone homeostasis through mechanosensation^6^,^7^,^8^,^9^. However, the mechanism of those proteins in regulating OB differentiation and function during skeletal development remains largely unknown.

Emerging evidence demonstrated that the mutations of cilia-related proteins such as polycystin-1 (Pkd1, a large membrane-spanning glycoprotein) and Kif3a (a member of kinesin family) in OPCs result in impaired OB differentiation and osteopenia^10^,^11^. Further studies have shown that conditional deletion of *Pkd1* or *Kif3a* in mature OBs impairs OB sensory function and disrupts mechanosensation-mediated skeletal homeostasis^8^,^12^. Despite the new insights on these ciliary proteins, the underlying molecular mechanisms of IFT proteins remain unclear.

Recent studies have shown that mutations of IFT and other cilia-related proteins significantly affect Hh canonical signalling, but display a variety of phenotypes, indicating that individual IFT protein plays different roles in the regulation of Hh signalling^13^,^14^,^15^,^16^. Primary cilium provides a specialized environment hosting the key components of the Hh canonical signal pathway, including Hh receptor Patched 1 (Ptch1), Smoothened (Smo) protein—a G protein coupled receptor (GPCR) and Gli transcription factors^17^,^18^. When Hh is present and binds to Ptch1, Ptch1 releases its inhibition on Smo and allows Smo to translocate into cilia. In cilia, active Smo causes the dissociation of Gli from Suppressor of Fused (SUFU), which allows Gli to be processed into its activated form^19^. In OBs, activated Gli regulates *BMP2* and *Runx2* expression^20^,^21^, which are critical regulators of OB differentiation and bone formation^20^,^22^,^23^. Therefore, Hh–Gli signalling plays the critical role in OB differentiation and bone development^24^,^25^. Mice lacking Shh fail to form vertebrae and limbs due to the inhibition of endochondral ossification^25^, confirming that Shh is indispensable for bone formation. However, the most recent evidence has shown that not all Hh signalling goes through Gli activation, the canonical Hh signalling pathway^26^,^27^. Gli-independent Hh signalling is recognized and referred to as the non-canonical Hh signalling. One of the non-canonical Hh signalling activates small GTPases RhoA and Rac1 via Smo-coupled Gαi proteins and regulates actin cytoskeleton in fibroblasts and endothelial cells^28^,^29^,^30^. Nevertheless, whether and how non-canonical Hh signalling regulates OB differentiation is unknown.

IFT80 is an IFT protein in IFT complex B. Mutations with reduced expression of *IFT80* in human cause Jeune asphyxiating thoracic dystrophy (JATD) and short rib polydactyly type III (SRPIII). Both diseases have bone abnormalities including shortening of the long bones and constriction of the thoracic cage^31^,^32^,^33^. JATD and SRPIII often lead to death in the prenatal stage or during infancy due to respiratory insufficiency. However, it is still unknown about the role and mechanism of IFT80 in bone development and OB differentiation. Previously, we found that IFT80 regulates osteogenesis through Hh–Gli signalling *in vitro*^34^. In this study, by using *IFT80* conditional knockout model, we further found that IFT80 is essential for balancing canonical and non-canonical Hh signalling pathways in OB differentiation. Deletion of *IFT80* disrupts canonical Hh–Gli activation but overactivates non-canonical Hh–Gαi–RhoA–ROCK (Rho-associated coiled-coil-containing kinases) pathway through altering Smo ciliary location and increasing Gαi and Smo binding. Inhibition of RhoA or ROCK promotes ciliogenesis and treatment of *IFT80*-deficient OPCs with cytochalasin D (CytoD), a filamentous actin (F-actin) destabilizer, greatly restores OB differentiation through promoting canonical Hh–Gli transduction and inhibiting Hh–RhoA–stress fibre non-canonical signalling. These findings reveal dual regulation of IFT80 in Hh signalling. On one hand, IFT80 promotes Hh canonical signalling via activation of Hh–Smo–Ptch1–Gli signalling pathway; on the other hand, IFT80 inhibits Hh non-canonical signalling via Hh–Smo–Gαi–RhoA–stress fibre signalling in OB differentiation.

## Results

### *IFT80* deletion causes growth retardation and osteopenia

**Figure 1:**
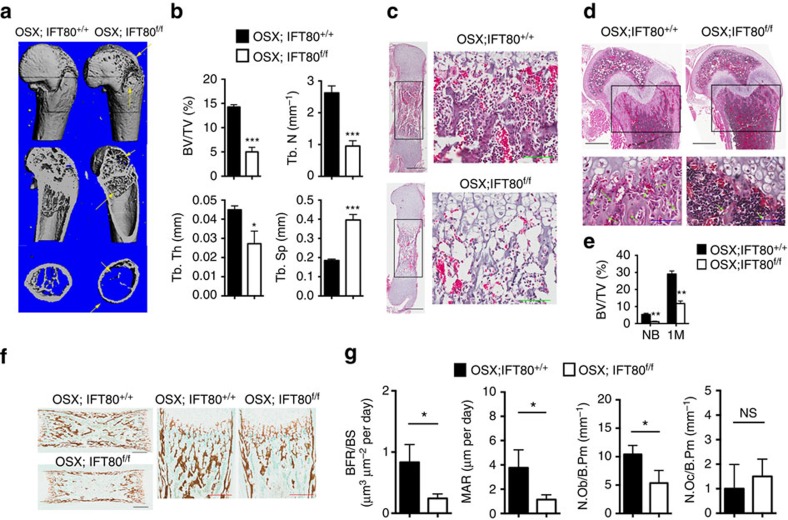
Deletion of *IFT80* causes a significant growth retardation and osteopenia. (**a**) Micro-CT of the femurs from 1-month-old *OSX; IFT80*^*+/+*^ mice and *OSX;IFT80*^*f/f*^ mice. Yellow arrows indicate the bone defects. (**b**) Quantitative analysis of the percentage of bone volume to total bone volume (BV/TV), trabecular thickness (Tb.Th), trabecular number (Tb.N), and trabecular spacing (Tb.Sp) in the femurs of 1-month-old *OSX; IFT80*^*+/+*^ mice and *OSX;IFT80*^*f/f*^ mice (*n*=4 mice per group). (**c**) Histological H&E staining analysis of newborn mice tibia. Right panel shows higher magnification of trabecular bone area. Scale bars, 500 μm (left, black) or 100 μm (right, green) (**d**) Histological H&E staining analysis of 1-month mice tibia. Lower panel shows higher magnification of trabecular bone area. Scale bars, 500 μm (top, black) or 100 μm (bottom, blue) (**e**) Quantitative analysis of the percentage of bone area to total bone marrow space in tibia (black rectangle) of *OSX; IFT80*^*+/+*^ mice and *OSX;IFT80*^*f/f*^ mice shown in **c**,**d** (*n*=4 mice per group). (**f**) Von Kossa staining of newborn mice tibia. Scale bars, 500 μm (left, black) or 200 μm (right, red). (**g**) Quantification of mineral apposition rate per bone surface (MAR/BS), bone formation rate (BFR), OB number per bone perimeter (Ob.N/B.Pm) and osteoclast number per bone perimeter (Oc.N/B.Pm) from double calcein injection experiment (*n*=3 mice per group). Error bars, s.e.m.; *P<0.05, **P<0.01 and ***P<0.0001 as determined by Student's *t*-test. NS, not statistically significant.

The mouse *IFT80* gene spans 142,546 bp of genomic DNA and contains 19 exons on chromosome 3. We generated *IFT80*^*flox*^ mice that contain two loxP sites flanking exon 6, as described in Methods (Supplementary Fig. 1a). Homozygous *IFT80*^*f/f*^ mice exhibited normal lifespan and fertility without any apparent phenotypic abnormality. Genomic DNA from tails was used for PCR genotyping. The 469-bp band corresponds to *IFT80*^*flox*^ and the 247-bp band corresponds to wild-type (*IFT80*^*+*^) (Supplementary Fig. 1b). To understand the role of IFT80 in bone development, we generated *OSX-Cre;IFT80*^*f/f*^ mice (referred to as *OSX;IFT80*^*f/f*^) by crossing *IFT80*^*f/f*^ mice with *OSX-Cre* transgenic mice^35^. Western blot analysis confirmed that the expression of IFT80 was significantly decreased in the calvarial bone of *OSX;IFT80*^*f/f*^ mice (Supplementary Fig. 1c). Quantitative PCR (QPCR) analysis showed that *IFT80* expression was almost absent from OPCs derived from the calvarial bone of *OSX;IFT80*^*f/f*^ mice (Supplementary Fig. 1d). *OSX;IFT80*^*f/f*^ mice were born with expected Mendelian ratios, but showed growth retardation (Supplementary Fig. 2a) accompanied with significantly decreased body weight starting at postnatal day 5 (Supplementary Fig. 2b). In addition, *OSX;IFT80*^*f/f*^ mice showed shorter body length starting from 1 month old (Supplementary Fig. 2c). Tibiae length of newborn *OSX;IFT80*^*f/f*^ mice were slightly shorter than that of *OSX;IFT80*^*+/+*^ mice, however, tibiae length of 4-month old *OSX;IFT80*^*f/f*^ mice were significantly shorter than that of *OSX;IFT80*^*+/+*^ mice (Supplementary Fig. 2d). Microcomputed tomography (Micro-CT) results of femurs from one-month-old mice showed an apparent decrease in the bone mass of *OSX;IFT80*^*f/f*^ mice in both trabecular and cortical bone with apparent bone defects (Fig. 1a). The percentage of bone volume to total bone volume (BV/TV), trabecular thickness (Tb.Th) and trabecular number (Tb.N) of *OSX;IFT80*^*f/f*^ mice were, respectively, 0.34-, 0.35- and 0.55-fold of that in *OSX;IFT80*^*+/+*^ mice (Fig. 1b). Trabecular spacing (Tb.Sp) of *OSX;IFT80*^*f/f*^ mice was almost twofold of that in *OSX;IFT80*^*+/+*^ mice (Fig. 1b). These data suggest an osteopenia phenotype. Moreover, *OSX;IFT80*^*f/f*^ mice also displayed narrow rib cages (Supplementary Fig. 2e) that mimic the patients with *IFT80* mutations.

To further confirm the phenotype and fully understand the role of IFT80 in skeletal development, histological haematoxylin and eosin (H&E) staining analysis was performed in the tibiae section of *OSX;IFT80*^*f/f*^ mice. The tibiae from newborn and 1-month-old *OSX;IFT80*^*f/f*^ mice all showed decreased trabecular bone (Fig. 1c,d). The difference in percentage of bone area to total marrow space in long bones of *OSX;IFT80*^*f/f*^ mice was 0.32-fold of that in *OSX;IFT80*^*+/+*^ mice at birth, and 0.41-fold of that at 1 month (Fig. 1e). Von Kossa staining of tibiae from newborn mice confirmed the reduced mineralization in *OSX;IFT80*^*f/f*^ group (Fig. 1f). Dynamic histomorphometric analyses demonstrated a significant reduction in mineral apposition rate and bone formation rate in *OSX;IFT80*^*f/f*^ group compared with *OSX;IFT80*^*+/+*^ group (Fig. 1g).

OB number per bone perimeter (B.Pm) was significantly reduced in the *OSX;IFT80*^*f/f*^ tibiae, while the osteoclast number per B.Pm had no change compared with *OSX;IFT80*^*+/+*^ group (Fig. 1g). Histological analysis showed elongated growth plate especially hypertrophic cartilage in *OSX;IFT80*^*f/f*^ mice compared with *OSX; IFT80*^*+/+*^ mice (Supplementary Fig. 2f). Secondary ossification was well-developed in the femurs and tibiae of *OSX;IFT80*^*+/+*^ mice, but was greatly delayed in *OSX;IFT80*^*f/f*^ mice at postnatal day 14 (Supplementary Fig. 2g).

### Deletion of *IFT80* blocks OB differentiation

**Figure 2:**
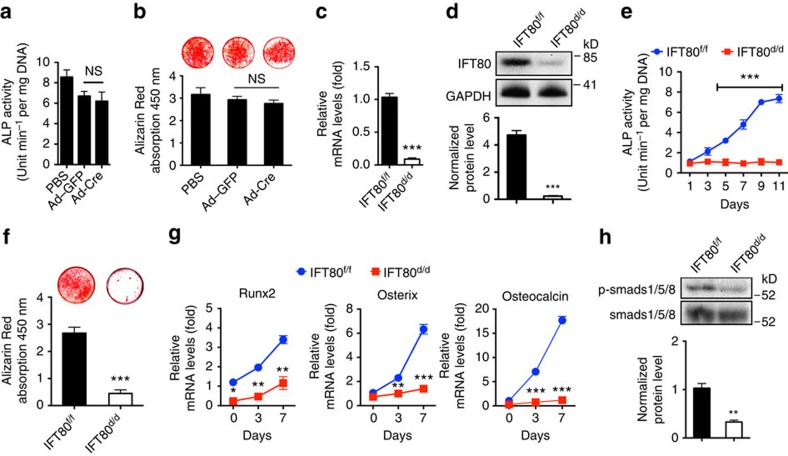
Deletion of *IFT80* impairs OB differentiation. (**a**) ALP activity of wild-type OPCs treated with Ad–GFP or Ad-Cre at D7 of osteogenic induction (*n*=3, triplicates per group). (**b**) Alizarin Red staining of wild-type OPCs treated with Ad–GFP or Ad-Cre at D21 of osteogenic induction (*n*=3, triplicates per group). (**c**) QPCR analysis of *IFT80* expression in *IFT80*^*f/f*^ and *IFT80*^*d/d*^ OPCs. The expression of *IFT80* is normalized to *GAPDH* expression (*n*=4, triplicates per group). (**d**) Western blot analysis of IFT80 expression in *IFT80*^*f/f*^ and *IFT80*^*d/d*^ OPCs. IFT80 protein level is normalized to GAPDH (*n*=3). (**e**) ALP activity of *IFT80*^*f/f*^ and *IFT80*^*d/d*^ OPCs during osteogenesis at indicated time points (*n*=4, triplicates per group). (**f**) Alizarin Red staining of *IFT80*^*f/f*^ and *IFT80*^*d/d*^ OPCs induced with OS medium for 21 days (*n*=3, triplicates per group). (**g**) QPCR analysis for OB marker genes *Runx2*, *osterix* and *osteocalcin* in OS culture medium at day 0, day 3 and day 7 in *IFT80*^*f/f*^ and *IFT80*^*d/d*^ groups (*n*=4, triplicates per group). (**h**) Western blot analysis of p-Smads 1/5/8 expression in *IFT80*^*f/f*^ and *IFT80*^*d/d*^ OPCs. p-Smads 1/5/8 protein level was normalized to Smads 1/5/8 (*n*=3 wells per group). Data were analysed using an appropriate Student's *t*-test, one-way ANOVA, or two-way analysis of variance followed by *post hoc* Tukey's analysis. Error bars, s.e.m.; *P<0.05, **P<0.01, ***P<0.0001. NS, not statistically significant.

To determine whether the decreased bone mass phenotype of *OSX;IFT80*^*f/f*^ mice resulted from impaired OBs, we studied the role of IFT80 in OB differentiation, function and proliferation *in vitro*. Primary OPCs were isolated from *IFT80*^*f/f*^ mice, and infected with Ad-CMV-Cre to delete *IFT80* as described in Methods (referred to as *IFT80*^*d/d*^). Ad–green fluorescent protein (Ad–GFP)-infected *IFT80*^*f/f*^ cells were used as control and still marked as *IFT80*^*f/f*^. Ad–GFP infection showed similar osteogenesis as Ad-CMV-Cre infection in wild-type OBs as detected by ALP activity and Alizarin Red staining (Fig. 2a,b), suggesting Ad-CMV-Cre has no significant effect on OB differentiation compared with Ad–GFP. Ad–GFP-infected *IFT80*^*f/f*^ cells showed higher GFP signalling in the beginning of osteogenesis but reduced at day 14 of OS induction (Supplementary Fig. 3a,b). Western blot and QPCR showed that Ad-CMV-Cre transduction yielded 95% reduction of IFT80 in *IFT80*^*f/f*^ OPCs (Fig. 2c,d). Deletion of *IFT80* in OPCs impaired OB differentiation and maturation as shown with less ALP activity (Fig. 2e), reduced Alizarin Red accumulation (Fig. 2f) and lower expression level of OB differentiation markers, including *Runx2*, *osterix* and *osteocalcin* (Fig. 2g). Compared with that in *IFT80*^*f/f*^ OBs, phosphorylation of Smads 1/5/8 in *IFT80*^*d/d*^ OBs was significantly reduced (Fig. 2h). These data are consistent with the bone defect phenotypes observed in *OSX;IFT80*^*f/f*^ mice and highlight the important role of IFT80 in OB differentiation. However, we cannot simply rule out the role of IFT80 in OB proliferation. Ki67 staining and MTS assay were performed to study cell proliferation and the data showed that deletion of IFT80 also inhibited the OB proliferation (Supplementary Fig. 4a,b). Hh agonist purmorphamine did not promote cell proliferation in both *IFT80*^*f/f*^ and *IFT80*^*d/d*^ groups (Supplementary Fig. 4b). To eliminate the proliferation effect on cell differentiation, we performed all the differentiation-related assays under the condition that the cells have reached confluency as both *IFT80*^*f/f*^ and *IFT80*^*d/d*^ showed similar and low-proliferation rate (Supplementary Fig. 4c).

### *IFT80* deletion disrupts ciliogenesis and Hh–Gli signalling

**Figure 3:**
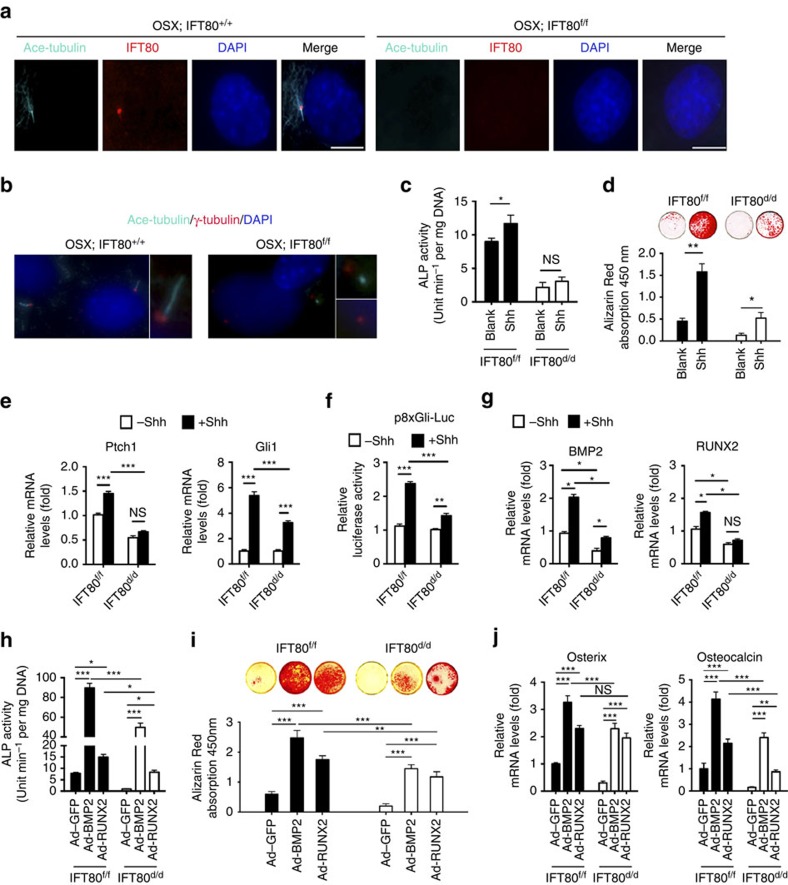
Deletion of *IFT80* disrupts cilia formation and inhibits canonical Hh–Gli signalling transduction in OPCs. (**a**) Immunofluorescence analysis of IFT80 in *IFT80*^*f/f*^ and *IFT80*^*d/d*^ OPCs. IFT80 (red) was co-stained with acetylated tubulin (ciliary axoneme, cyan). DAPI (nuclear marker) staining is used as counterstaining. Scale bars, 10 μm. (**b**) Immunofluorescence analysis of primary cilia in *IFT80*^*f/f*^ and *IFT80*^*d/d*^ OPCs. Primary cilia were stained with γ-tubulin (basal body, red) and acetylated α-tubulin (axoneme, cyan) antibody. The inset shows a high-power image of the basal body and axoneme. DAPI staining is used as counterstaining. Scale bars, 10 μm. (**c**) ALP activity of *IFT80*^*f/f*^ and *IFT80*^*d/d*^ OPCs at day 7 of OS induction with or without Shh stimulation (*n*=4, triplicates per group). (**d**) Alizarin Red of *IFT80*^*f/f*^ and *IFT80*^*d/d*^ OPCs at day 14 of OS induction with or without Shh stimulation (*n*=3, triplicates per group). (**e**) QPCR results showing *Ptch1* and *Gli1* expression in *IFT80*^*f/f*^ and *IFT80*^*d/d*^ OPCs with or without Shh stimulation (*n*=4, triplicates per group). (**f**) Reporter assay showing 8 × Gli responsive luciferase (p8 × Gli-Luc) activity in *IFT80*^*f/f*^ and *IFT80*^*d/d*^ OPCs with or without Shh stimulation (*n*=3, triplicates per group). (**g**) QPCR results showing *BMP2* and *Runx2* expression in *IFT80*^*f/f*^ and *IFT80*^*d/d*^ OPCs with or without Shh stimulation (*n*=4, triplicates per group). (**h**) ALP activity of *IFT80*^*f/f*^ and *IFT80*^*d/d*^ OPCs transfected with BMP2 or RUNX2 adenovirus. Both cells were induced for osteogenesis for 7 days (*n*=4, triplicates per group). (**i**) Alizarin Red staining at day 14 in OS culture. *IFT80*^*f/f*^ and *IFT80*^*d/d*^ OPCs were transfected with BMP2 or Runx2 adenovirus (*n*=4, triplicates per group). (**j**) QPCR result showing *osterix* and *osteocalcin* expression with BMP2 or Runx2 adenovirus transfection in *IFT80*^*f/f*^ and *IFT80*^*d/d*^ group (*n*=4, triplicates per group). Data points indicate the means, while error bars represent s.e.m. Data were analysed using two-way ANOVA followed by Tukey's multiple comparison test. *P<0.05, **P<0.01, ***P<0.0001. NS, not statistically significant.

To further confirm that *IFT80* was deleted after Ad-CMV-Cre infection, we performed immunofluorescence staining for IFT80 in OPCs from *OSX;IFT80*^*+/+*^ and *OSX;IFT80*^*f/f*^ mice. In *OSX;IFT80*^*+/+*^ OPCs, IFT80 protein was detected in the basal bodies and along the ciliary axoneme. However, IFT80 was absent in *OSX;IFT80*^*f/f*^ OPCs (Fig. 3a). To further analyse the role of IFT80 in ciliogenesis, we performed double staining of the basal body (anti-γ tubulin) and axoneme (anti-acetylated α-tubulin) to visualize cilia. About 80% of the cells in *OSX;IFT80*^*+/+*^ group have normal cilia structure, while there were substantially fewer ciliated cells in *OSX;IFT80*^*f/f*^ group. More than 60% of the cells lost cilia structure and only the centrosomes were stained in *OSX;IFT80*^*f/f*^ group. About 30% of the cells had cilia structure but displayed shortened cilia compared with *OSX;IFT80*^*+/+*^ group (Fig. 3b). These data indicate that IFT80 is essential for cilia formation.

To explore the mechanisms by which loss of IFT80 blocks OB differentiation, we characterized the effect of *IFT80* deletion on Hh–Gli canonical signalling pathway. Treatment of *IFT80*^*f/f*^ OPCs with Shh (1 μg ml^−1^) resulted in a significant promotion in OB differentiation (Fig. 3c,d) and a significant increase in the expression of *Gli1* and *Ptch1*, indicating the activation of Hh signalling (Fig. 3e). In contrast, Shh failed to similarly stimulate OB differentiation, elevate *Gli1* and *Ptch1* expression (Fig. 3c–e), and promote Gli-responsive-promoter luciferase activity in *IFT80*^*d/d*^ OPCs (Fig. 3f). Hh agonist purmorphamine also failed to stimulate OB differentiation in *IFT80*^*d/d*^ group (Supplementary Fig. 5a,b). These data suggest that loss of IFT80 impairs Hh–Gli signalling in OPCs. Consistent with previous reports that Hh signalling enhances Runx2 and BMP2 expression^20^,^21^, we found that Shh promoted *Runx2* and *BMP2* expression in *IFT80*^*f/f*^ OPCs (Fig. 3g) but failed to increase the expression of these genes in *IFT80*^*d/d*^ OPCs (Fig. 3g). These results demonstrate that deletion of *IFT80* blocks OB differentiation due to impaired cilia formation and Hh–Gli transduction.

Since BMP2 and Runx2 act downstream of Hh–Gli signalling, we tested whether overexpression of BMP2 or Runx2 could restore osteogenesis in *IFT80*^*d/d*^ OPCs. Infection of *IFT80*^*f/f*^ and *IFT80*^*d/d*^ OPCs with Ad-BMP2 or Ad-Runx2 significantly promoted *BMP2* or *Runx2* expression (Supplementary Fig. 6a,b). Overexpressing either BMP2 or Runx2 partially rescued osteogenesis in *IFT80*^*d/d*^ OPCs, as evidenced by restored ALP activity (Fig. 3h), Alizarin Red accumulation (Fig. 3i), and the expression of *osterix* and *osteocalcin* (Fig. 3j). However, overexpression of BMP2 or Runx2 could not fully restore *IFT80*^*d/d*^ OB differentiation, suggesting that a parallel mechanism might exist to cooperate with Hh–Gli pathway to regulate OB differentiation.

### *IFT80* deletion activates non-canonical Gαi–RhoA signalling

**Figure 4:**
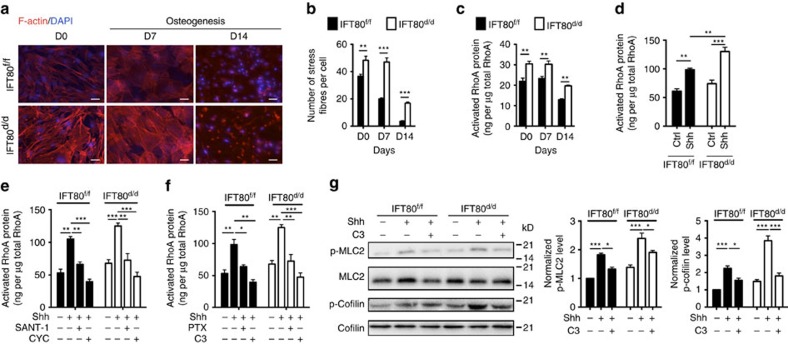
Deletion of *IFT80* elevates non-canonical Hh–RhoA signalling transduction in OPCs. (**a**) *IFT80*^*f/f*^ and *IFT80*^*d/d*^ OBs were stained to visualize actin (red) and nucleus (blue). Scale bars, 100 μm. (**b**) Graph quantifying the percentage of stress fibres shown in **a** (*n*=3 with at least 15 cells analysed). (**c**) Activated RhoA was detected by ELISA-based G-LISA assay during osteogenesis at indicated time points (*n*=4, triplicates per group). (**d**) G-LISA assay showing RhoA activation in *IFT80*^*f/f*^ and *IFT80*^*d/d*^ OPCs treated with Shh for 20 min (*n*=4, triplicates per group). (**e**) G-LISA assay showing RhoA activation in *IFT80*^*f/f*^ and *IFT80*^*d/d*^ OPCs. Both OPCs were treated with Smo inhibitors SANT-1 and cyclopamine (CYC) and then stimulated with Shh for 20 min (*n*=4, triplicates per group). (**f**) G-LISA assay showing RhoA activation in *IFT80*^*f/f*^ and *IFT80*^*d/d*^ OPCs. Both OPCs were treated with PTX (Gαi inhibitor) or C3 (RhoA inhibitor) and then stimulated with Shh for 20min (*n*=4, triplicates per group). (**g**) Western blot analysis of phospho-MLC2 (p-MLC2) and phospho-Cofilin (p-Cofilin) in *IFT80*^*f/f*^ and *IFT80*^*d/d*^ OPCs. The levels of p-MLC2 and p-Cofilin are normalized to total MLC2 and Cofilin (*n*=3). Data points indicate the means, while error bars represent s.e.m. Data were analysed using two-way ANOVA followed by Tukey's multiple comparison test. *P<0.05, **P<0.01, ***P<0.0001.

Given that cilia are associated with actin cytoskeleton^36^, we analysed actin stress fibre density and found that deletion of *IFT80* significantly increased stress fibre density (Fig. 4a,b). RhoA is a small GTPase protein, which regulates actin cytoskeleton^37^. Therefore, we measured RhoA activity during OB differentiation. Consistent with the stress fibre results, RhoA activity was significantly reduced along the differentiated progression of both *IFT80*^*f/f*^ and *IFT80*^*d/d*^ OBs (Fig. 4c). However, *IFT80*^*d/d*^ OBs showed significantly higher RhoA activity than *IFT80*^*f/f*^ OBs during OB differentiation (Fig. 4c).

Non-canonical Hh signalling pathway has been reported in endothelial cells, which regulates actin stress fibres in response to Hh via a Gli-independent mechanism^30^. Since Hh–Gli transduction was blocked and RhoA activity and stress fibre were increased in *IFT80*^*d/d*^ OPCs, we hypothesized that IFT80 regulates RhoA–stress fibre signalling in a Hh-dependent manner and that loss of IFT80 shifts activation of Hh signalling from the canonical to non-canonical transduction thereby inhibiting OB differentiation. To test this hypothesis, we first determined whether the effect of IFT80 on stress fibre and RhoA activity is Hh-dependent in OBs. We found RhoA activity increased 1.5-fold in *IFT80*^*f/f*^ OPCs with Shh stimulation for 20 min, suggesting that OPCs can respond rapidly to Shh via the Hh non-canonical pathway (Fig. 4d). RhoA activity was greatly promoted by Shh and increased by 1.9-fold in *IFT80*^*d/d*^ OPCs. Thus, these results demonstrated that deletion of *IFT80* overactivates the non-canonical Hh–RhoA signalling pathway. To get further insight into the mechanism, we then tested whether this non-canonical Hh–RhoA signalling transduction is Smo dependent by using Smo inhibitors—SANT-1 and cyclopamine. Consistent with other studies^38^,^39^, SANT-1 and cyclopamine significantly inhibited Hh–Gli signalling transduction in both *IFT80*^*f/f*^ and *IFT80*^*d/d*^ OPCs (Supplementary Fig. 7a). Both SANT-1 and cyclopamine inhibited RhoA activity in response to Shh stimulation (Fig. 4e), indicating that Hh–RhoA transduction is Smo dependent. To further confirm this conclusion, C3 (RhoA inhibitor) and Pertussis toxin (PTX, Gαi inhibitor) were used to inhibit RhoA and its upstream regulator—Gαi in non-canonical Hh signalling pathway. The results showed that PTX and C3 significantly dampened RhoA activation induced by Shh (Fig. 4f). ROCK, a downstream effector of RhoA^40^, inactivates Cofilin, an actin depolymerizing protein, by phosphorylation. ROCK promotes MLC2 phosphorylation through inactivating MLC phosphatase. Both downstream effectors of ROCK regulate the stress fibre formation^41^. Since RhoA activity was overactivated in *IFT80*^*d/d*^ OPCs, we measured the phosphorylation of MLC2 and Cofilin. As we expected, without Shh stimulation, there was no significant difference in phosphorylation of MLC2 and Cofilin between *IFT80*^*f/f*^ and *IFT80*^*d/d*^ OPCs. In contrast, the phosphorylation levels were significantly increased in *IFT80*^*d/d*^ OPCs with Shh stimulation (Fig. 4g). Moreover, inhibition of RhoA activity by C3 significantly inhibited Shh-induced phosphorylation of MLC2 and Cofilin (Fig. 4g). Collectively, these results suggest that deletion of *IFT80* overactivates non-canonical Hh signalling pathway showing increasing RhoA activity and stress fibre formation.

### Inhibition of non-canonical Hh signalling promotes osteogenesis

**Figure 5:**
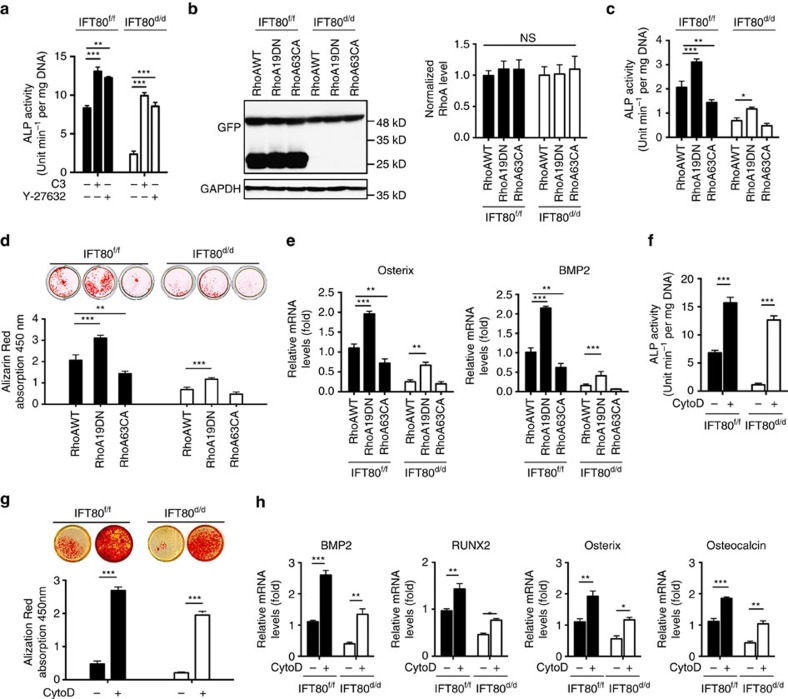
Inhibition of non-canonical Hh signalling promotes osteogenesis. (**a**) ALP activity assay of *IFT80*^*f/f*^ and *IFT80*^*d/d*^ OPCs at day 7 of osteogenic induction. Both OPCs were treated with 0.5 μg ml^−1^ C3 (RhoA inhibitor) or 10 μM Y-27632 (ROCK inhibitor) (*n*=4, triplicates per group). (**b**) Western blot of GFP showing the expression level of GFP-tagged RhoA proteins in different groups. The bands of 27 kDa represent the GFP proteins in the *IFT80*^*f/f*^ group that were transfected with Ad–GFP. The bands of 48 kDa represent the GFP-tagged RhoA. The levels of RhoAWT, RhoA19DN and RhoA63CA are normalized to GAPDH (*n*=3, NS, not statistically significant). (**c**) ALP activity assay of *IFT80*^*f/f*^ and *IFT80*^*d/d*^ OPCs transfected with RhoA19DN (dominant negative) and RhoA63CA (constitutively active) at day 7 in OS culture (*n*=3, triplicates per group). (**d**) Alizarin Red staining showing the OB mineralization of *IFT80*^*f/f*^ and *IFT80*^*d/d*^ OPCs at day 14 in OS culture with RhoA19DN and RhoA63CA transfection (*n*=3, triplicates per group). (**e**) QPCR shows *Osterix* and *BMP2* expression at day 7 in OS culture in *IFT80*^*f/f*^ and *IFT80*^*d/d*^ OPCs, which were transfected with RhoA19DN and RhoA63CA (*n*=3, triplicates per group). (**f**) ALP activity assay of *IFT80*^*f/f*^ and *IFT80*^*d/d*^ OPCs at day 7 of OS induction with or without CytoD treatment (*n*=4, triplicates per group). (**g**) Alizarin Red staining showing the OB mineralization of *IFT80*^*f/f*^ and *IFT80*^*d/d*^ OPCs at day 14 in OS culture with or without CytoD treatment (*n*=4, triplicates per group). (**h**) QPCR shows *BMP2*, *Runx2*, *osterix* and *osteocalcin* expression in *IFT80*^*f/f*^ and *IFT80*^*d/d*^ OPCs with or without CytoD treatment. Data points indicate the means, while error bars represent s.e.m. Data were analysed using two-way ANOVA followed by Tukey's multiple comparison test. *P<0.05, **P<0.01, ***P<0.0001.

To test whether elevated Hh–RhoA activity in *IFT80*^*d/d*^ cells inhibits OB differentiation, RhoA inhibitor—C3 and ROCK inhibitor—Y-27632 were used to suppress RhoA and ROCK activation. Treatment of *IFT80*^*d/d*^ OPCs with C3 or Y-27632 greatly restored ALP activity (Fig. 5a), suggesting that overactivated RhoA signalling is another cause of defective OB differentiation in *IFT80*^*d/d*^ OPCs. To further confirm the role of RhoA in OB differentiation, *IFT80*^*f/f*^ and *IFT80*^*d/d*^ OPCs were stably transfected with dominant negative RhoA (RhoA19DN) and constitutively active RhoA (RhoA63CA) vectors and then induced for OB differentiation. Western blot confirmed the similar expression level of RhoA19DN and RhoA63CA in both *IFT80*^*f/f*^ and *IFT80*^*d/d*^ OPCs (Fig. 5b). As expected, dominant negative RhoA inhibited stress fibre formation while constitutively active RhoA promotes stress fibre formation (Supplementary Fig. 8a,b). Dominant negative RhoA promoted ALP activity, the formation of Alizarin Red-positive nodules and the expression of *osterix* and *BMP2* (Fig. 5c–e) in both *IFT80*^*f/f*^ and *IFT80*^*d/d*^ OPCs; however, the expression of dominant negative RhoA partially restored OB differentiation in *IFT80*^*d/d*^ OPCs, suggesting that RhoA–stress fibre pathway might be a parallel pathway to cooperate with Hh–Gli pathway to regulate OB differentiation.

CytoD is an F-actin destabilizer by binding to the plus end of F-actin to block the polymerization and the elongation of actins^42^,^43^. CytoD treatment significantly restored ALP activity and greatly promoted Alizarin Red accumulation in the extracellular matrix in both *IFT80*^*f/f*^ and *IFT80*^*d/d*^ OPCs (Fig. 5f,g). CytoD treatment also markedly promoted *BMP2*, *Runx2*, *osterix* and *osteocalcin* expression in both *IFT80*^*f/f*^ and *IFT80*^*d/d*^ OPCs (Fig. 5h), indicating the increase of stress fibres in *IFT80*^*d/d*^ OPCs inhibits OB differentiation.

### Inhibition of non-canonical Hh promotes ciliogenesis

**Figure 6:**
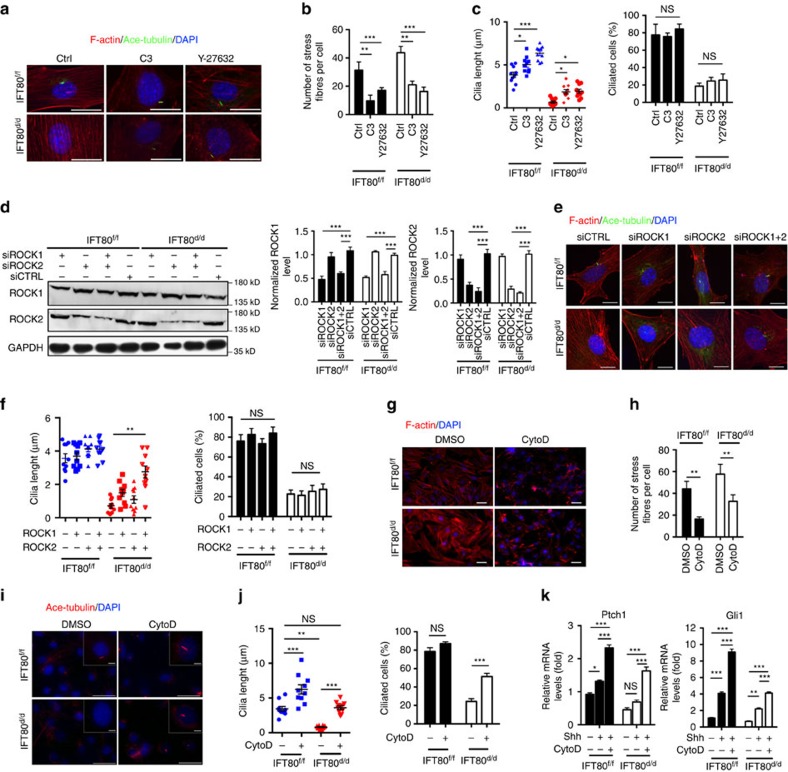
Inhibition of non-canonical Hh signalling reduces stress fibre density while promotes ciliogenesis. (**a**) *IFT80*^*f/f*^ and *IFT80*^*d/d*^ OPCs were stained for actin (red) and cilia (green) after C3 (RhoA inhibitor) or Y-27632 (ROCK inhibitor) treatment. DAPI staining is used as counterstaining. Scale bars, 25 μm. (**b**) Graph quantifying the percentage of stress fibres shown in **a** (*n*=3 with at least 15 cells analysed). (**c**) Calculated cilia length (*n*=10 cells) and cilia percentage (*n*=3 with at least 200 cells analysed) shown in **a**. (**d**) Western blot results showing ROCK1 and ROCK2 expression in *IFT80*^*f/f*^ and *IFT80*^*d/d*^ OPCs after transfected with control siRNA (siCTRL) or siRNA targeting ROCK1, siROCK2 or both. The level of ROCK1 and ROCK2 expression are normalized to GAPDH (*n*=3). (**e**) *IFT80*^*f/f*^ and *IFT80*^*d/d*^ OPCs were stained with stress fibre (red), cilia (green) and the nucleus (blue) after transfected with control siRNA (siCTRL) or siRNA targeting ROCK1, siROCK2 or both. Scale bars, 25 μm. (**f**) Calculated cilia length (*n*=10 cells) and cilia percentage (*n*=3 with at least 200 cells analysed) shown in **e**. (**g**) *IFT80*^*f/f*^ and *IFT80*^*d/d*^ OPCs were stained for actin (red) after CytoD treatment. DAPI staining is used as counterstaining. Scale bars, 100 μm. (**h**) Graph quantifying the number of stress fibres shown in **g** (*n*=3 with at least 15 cells analysed). (**i**) Immunofluorescence analysis of cilia (acetylated α-tubulin, red) in *IFT80*^*f/f*^ and *IFT80*^*d/d*^ OPCs with or without CytoD treatment. The insets show a high-power image of cilia. DAPI staining was used as counterstaining. Scale bars, 50 μm. (**j**) Calculated cilia length (*n*=3 with at least 10 cells measured) and ciliated cell percentage (*n*=3 with at least 200 cells analysed) shown in **i**. (**k**) QPCR showing *Gli1* and *Ptch1* expression in *IFT80*^*f/f*^ and *IFT80*^*d/d*^ OPCs with CytoD and Shh treatment (*n*=4, triplicates per group). Data points indicate the means, while error bars represent s.e.m. Data were analysed using two-way ANOVA followed by Tukey's multiple comparison test. *P<0.05, **P<0.01, ***P<0.0001. NS, not statistically significant.

Several studies have shown that actin cytoskeleton regulates ciliogenesis as actin cytoskeleton destabilization promotes ciliogenesis^42^,^44^. Therefore, we hypothesized that inhibition of non-canonical Hh–RhoA–stress fibre pathway by disrupting stress fibre could restore the ciliogenesis and rescue canonical Hh–Gli signalling transduction in *IFT80*^*d/d*^ OPCs. As expected, the cells treated with C3 and Y27632 showed a significantly reduction of stress fibres in both *IFT80*^*f/f*^ and *IFT80*^*d/d*^ OPCs (Fig. 6a,b). Although the percentage of ciliated cells was not significantly increased in C3 and Y27632 treated group, the cilia length is increased along with the actin destabilization (Fig. 6a,c). Even in *IFT80*^*d/d*^ OPCs group, those ciliated cells elongated in response to the C3 and Y27632 treatment. To further study the relationship between ciliogenesis and non-canonical Hh signalling, siRNAs were used to inhibit ROCK1 and ROCK2 expression in OPCs (Fig. 6d). siRNAs targeted against ROCK1 or ROCK2 alone slightly promoted cilia elongation; however, siRNA target both ROCK1 and ROCK2 greatly restored the cilia formation in *IFT80*^*d/d*^ OPCs (Fig. 6e,f), suggesting both ROCK1 and ROCK2 are involved in actin cytoskeleton related cilia formation. Some evidence showed that the perturbation of actin dynamics by CytoD promotes ciliogenesis and cilia elongation^45^,^46^. *IFT80*^*f/f*^ and *IFT80*^*d/d*^ OPCs were then treated with CytoD for 1 h, and stained for F-actin and cilia. The results showed that stress fibres were disrupted in both *IFT80*^*f/f*^ and *IFT80*^*d/d*^ OPCs (Fig. 6g,h). CytoD treatment led to a marked elongation of cilia in *IFT80*^*f/f*^ group (Fig. 6i,j). Moreover, CytoD treatment partially, but significantly, rescued defective ciliogenesis in *IFT80*^*d/d*^ OPCs (Fig. 6i). The average cilia length in DMSO-treated *IFT80*^*f/f*^ OPCs was 3.15 μm, while CytoD treatment increased the cilia length to 6.24 μm (Fig. 6j). In *IFT80*^*d/d*^ group, CytoD treatment increased cilia length from 0.8 to 3.6 μm (Fig. 6j). CytoD treatment did not significantly alter the percentage of ciliated cells in *IFT80*^*f/f*^ group, but increased the percentage of ciliated cells from 23 to 51% in *IFT80*^*d/d*^ group (Fig. 6j). These data suggest that the disruption of stress fibre could rapidly promote cilia formation in *IFT80*^*d/d*^ OPCs.

Since CytoD partially disrupted stress fibres and restored cilia formation in *IFT80*^*d/d*^ OPCs, we next analysed whether canonical Hh–Gli signalling transduction could be significantly restored with CytoD treatment. CytoD combined with Shh greatly promoted *Ptch1* and *Gli1* expression in both *IFT80*^*f/f*^ and *IFT80*^*d/d*^ groups (Fig. 6k), indicating that Hh–Gli signalling transduction in *IFT80*^*d/d*^ OPC was partially rescued by CytoD treatment. Hence, disruption of stress fibres by CytoD in non-canonical Hh signalling pathway could enhance canonical Hh pathway activity through promoting ciliogenesis.

### Deletion *IFT80* disrupts the ciliary localization of Smo

**Figure 7:**
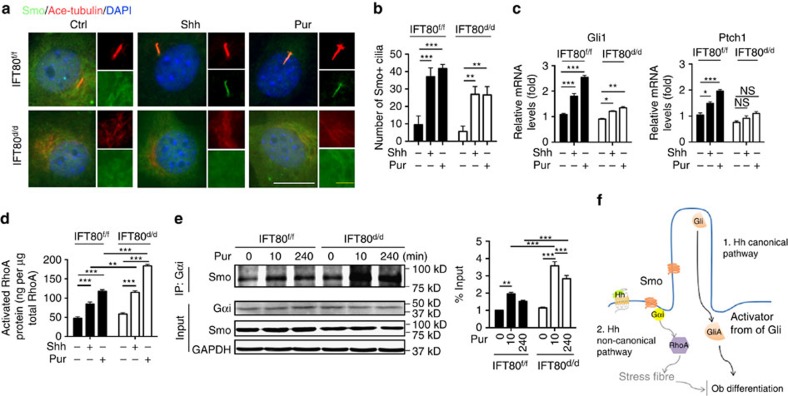
Defects of Smo ciliary localization in *IFT80*^*d/d*^ OPCs shifts the canonical Hh to non-canonical Hh transduction. (**a**) Immunostaining showing Smo localization (green) with Shh and purmorphomine (Pur) stimulation. Primary cilia were stained with acetylated α-tubulin antibody (red). DAPI staining is used as counterstaining. Scale bars, 15 μm (white) or 5 μm (yellow). (**b**) Quantification of GFP-positive cilia with or without Hh signalling activation shown in **a** (*n*=3 with at least 15 cells analysed). (**c**) QPCR results showing *Ptch1* and *Gli1* expression in both *IFT80*^*f/f*^ and *IFT80*^*d/d*^ OPCs with Shh or purmorphamine (Pur) stimulation (*n*=3, triplicates per group). (**d**) G-LISA assay showing that activated RhoA in *IFT80*^*f/f*^ and *IFT80*^*d/d*^ OPCs with Shh or purmorphamine (Pur) stimulation (*n*=4, triplicates per group). (**e**) Western blot showing the Gαi and Smo binding. *IFT80*^*f/f*^ and *IFT80*^*d/d*^ OPCs were treated with purmorphamine (Pur) for 10 min or 4 h, and cell lysates were used for immunoprecipitation and immunoblot with indicated antibodies. Smo binding was normalized to Smo levels in total lysates (*n*=3). Data points indicate the means, while error bars represent s.e.m. Data were analysed using two-way ANOVA followed by Tukey's multiple comparison test. *P<0.05, **P<0.01, ***P<0.0001. (**f**) Schematic representation of the proposed function of IFT80/cilia in canonical and non-canonical Hh signalling.

Since deletion of *IFT80* blocked canonical Hh signalling and overactivated non-canonical Hh signalling, and two pathways share Smo function, we next studied how IFT80 is involved in Smo translocation by immunofluorescence staining for Smo. The result showed that Shh or purmorphamine treatment led to Smo trafficking to cilia (Fig. 7a,b). Loss of IFT80 caused cilia loss and in turn resulted in the widespread cytoplasmic expression of Smo even with Shh or purmorphamine stimulation. Moreover, we found that in *IFT80*^*f/f*^ OPCs, Hh signalling activation elevated *Ptch1* and *Gli1* expression and promoted RhoA activation (Fig. 7c,d). However, in *IFT80*^*d/d*^ OPCs, Shh or purmorphamine slightly promoted *Ptch1* and *Gli1* expression but greatly activated RhoA compared with *IFT80*^*f/f*^ OPCs (Fig. 7c,d), suggesting ectopic activated Smo promotes both canonical and non-canonical Hh pathways, however, it favours non-canonical Hh–RhoA pathway under cilia absent condition. To further seek the reason of this preference, we tested whether the decrease of Smo translocation to cilia enhances Smo binding with Gαi. The results showed that purmorphamine stimulation increased the recruitment of Smo to Gαi binding in both *IFT80*^*f/f*^ and *IFT80*^*d/d*^ OPCs (Fig. 7e). However, the binding of Smo and Gαi were almost doubled in *IFT80*^*d/d*^ OPCs compared with *IFT80*^*f/f*^ with purmorphamine stimulation for 10 min or 240 min, respectively, suggesting the altered Smo ciliary translocation promotes the Smo and Gαi binding, which favors non-canonical Hh signalling activation in *IFT80*^*d/d*^ OPCs.

## Discussion

Although ciliary IFT proteins participate in the formation and homeostasis of various organs^3^, their roles in OB differentiation and bone development are not well-addressed. Our study reveals for the first time that non-canonical Hh signalling negatively regulates OB differentiation and that IFT80 is essential for the balance of the canonical and non-canonical Hh signalling pathways during OB differentiation and bone formation.

Interesting findings have showed that hypomorphic levels of IFT80 in mouse led to embryonic lethality associated with severe bone defects and disrupted Hh signalling^47^. However, it is unclear if the abnormal bone phenotype results from the direct effect of *IFT80* mutation on bone development and/or homeostasis or the indirect effect of *IFT80* mutation in other human tissues. Our results showed the mice with deletion of *IFT80 in* OPCs using *OSX-Cre* transgenic line shows growth retardation with dramatically decreased bone mass and impaired OB differentiation and proliferation, demonstrating that IFT80 directly affects OB differentiation, proliferation and bone development. In addition, weak OSX expression has been found in the hypertrophic chondrocytes^35^, thus, *IFT80* might also be deleted in part of the hypertrophic chondrocyte lineage in *OSX;IFT80*^*f/f*^ mice, contributing to the enlarged hypertrophic chondrocyte zone in growth plate. In our previous study, we have found that IFT80 is critical for chondrocytes differentiation by regulating Hh and Wnt signalling^48^,^49^. Deletion of *IFT80* in chondrocytes with inducible *Col2α1-Cre* causes reduced length of growth plate in both embryonic stage and postnatal stage^48^. The difference of cartilage phenotype observed in *OSX;IFT80*^*f/f*^ and *Col2α1;IFT80*^*f/f*^ mice might be due to the different expression pattern and level of *OSX* and *Col2α1* in cartilage. Therefore, the exact role of IFT80 in regulating cartilage formation needs further study.

Shh rapidly promoted RhoA activation within 20 min in both *IFT80*^*f/f*^ and *IFT80*^*d/d*^ OPCs via a Gαi-dependent pathway (Fig. 4d,f), while Hh-induced canonical Gli transduction response usually takes hours^29^, suggesting that Hh–Gαi–RhoA signalling occurs in OPCs, and that the rapid Hh–Gαi–RhoA signalling in OPCs is Gli-independent Hh signalling. Some studies have shown that this ligand-dependent non-canonical Hh signalling regulates actin cytoskeleton in endothelial cells, fibroblasts and cancer cell lines^29^,^30^,^50^,^51^,^52^, However, it is the first time that we identified this Gli-independent, but Smo-dependent non-canonical Hh signalling also exists in OPCs.

It has been reported that non-canonical Hh pathway participates in the chemotaxis of cholangiocarcinoma cells^52^, in the cytoskeletal rearrangement of mouse mesenchymal fibroblasts^50^, in the pro-angiogenic responses of endothelial cells^30^ and in the migration of fibroblast^29^. Here we uncovered an antagonistic role of non-canonical Hh–RhoA signalling pathway in OB differentiation. Deletion of *IFT80* elevated RhoA activity and downregulated BMP2-Smad1/5/8 signalling, which greatly inhibited OB differentiation. Inhibition of RhoA or its downstream effector ROCK restored ALP activity of *IFT80*^*d/d*^ OPCs. This is supported by the findings that activated RhoA signalling inhibits OB differentiation both *in vivo* and *in vitro*^53^,^54^. Although RhoA negatively regulates BMP2-induced Smad1/5/8 phosphorylation in a ROCK-independent way^55^, inhibition of ROCK activity by Y-27632 also promotes OB differentiation^54^,^56^,^57^. Activation of the non-canonical Hh pathway in endothelial cells promotes actin stress fibre formation^30^. Consistently, we found that overactivation of non-canonical Hh–RhoA signalling increased stress fibre density in OPCs (Fig. 4a,b).

Recently, a functional genomic screen using RNAi uncovered the regulatory role of actin cytoskeleton in ciliogenesis^42^. Inhibiting actin assembly promotes cilia formation by stabilizing the pericentrosomal preciliary compartment storing, which stores transmembrane proteins destined for cilia^42^. Inhibiting branched actin network formation by a microRNA mir-129-3p promotes ciliogenesis^58^. Very recently, actin destabilization has been shown to facilitate ciliogenesis by limiting deacetylation of tubulin in cilia^59^. Cytoskeleton remodelling also regulates transcriptional coactivator YAP/TAZ, which might serve as a key component of transcriptional networks governing ciliogenesis and cell proliferation^44^. Consistent with those studies, inhibition of RhoA, ROCK or actin stress fibre promotes ciliogenesis in our studies, suggesting that the relationship between stress fibre and cilia also existed in OPCs. The defective ciliogenesis in *IFT80*-deficient OPCs was partially rescued by cytoskeleton destabilization, which is possibly associated with the reduced cilia disassembly and increased soluble tubulin that facilitating the anterograde IFT under the *IFT80*-deletion caused defective anterograde IFT condition^45^. Notably, our results showed that CytoD also restored Hh–Gli transduction in *IFT80*-deficient OPCs (Fig. 6k), suggesting that non-canonical Hh–Gαi–RhoA–stress fibre pathway could feed back to Hh–Gli canonical signalling through remodelling the actin cytoskeleton. CytoD significantly rescued defective OB differentiation (Fig. 5f–h). This is likely because CytoD inhibited the stress fibre formation generated from Hh–RhoA activation, meanwhile restored the Hh–Gli canonical signalling in *IFT80*^*d/d*^ OPCs. This finding further confirmed that deletion of *IFT80* altered both canonical and non-canonical Hh pathways, which contribute to the defect in OB differentiation. Although our results indicate a role of CytoD in Hh–Gli signalling rescue, this does not exclude its involvement in other pathways. CytoD also activates protein kinase D, which is a potential pathway that promotes OB differentiation^60^.

The relationship between canonical Hh signalling and cilia is well-established—as effective Hh–Gli signalling transduction requires functional cilia^61^. The role of cilia in the non-canonical Hh signal transduction, however, is largely unknown. Our results showed that deletion of *IFT80* disrupted cilia formation but overactivated Hh–RhoA signalling, suggesting that this non-canonical signalling Hh pathway in OPCs is cilia independent. This novel concept is supported by the observation from Razumilava *et al.*^52^, where they found that malignant cholangiocarcinoma cells don't have cilia and purmorphamine induces remodelling of the actin cytoskeleton and migration in these cells through non-canonical Hh activation. We found that non-canonical Hh signalling was overactivated in *IFT80*^*d/d*^ OPCs with increased RhoA activity and stress fibre formation, while canonical Hh–Gli activity was disrupted. Bijlsma *et al.*^62^ found that mutation of ciliary localization motif in Smo favours the non-canonical Hh transduction in mouse embryonic fibroblasts, suggesting that subcellular localization of Smo determines how Hh signalling is transduced. Our results demonstrated that loss of *IFT80* favours the non-canonical Hh signalling transduction. In control cells, Smo localizes to cilia with Shh or purmorphamine stimulation (Fig. 7a,b), while loss of IFT80 caused cilia loss and in turn led to the widespread cytoplasmic expression of Smo even with Shh or purmorphamine stimulation. We further found that activation of Smo with purmorphamine could promote both *Ptch1* and *Gli1* expression, and RhoA activity in *IFT80*^*f/f*^ OPCs, however, it favours to activate non-canonical Hh–RhoA pathway in *IFT80*^*d/d*^ OPCs, which can be explained by increased binding of Smo with Gαi (Fig. 7e). Inevitably, these observations raise a series of important questions regarding the regulation of canonical Hh and non-canonical Hh signalling pathway. Whether Smo undergoes differential post-translational modification and activation within or outside cilia remains unknown. How Smo subcellular distribution is maintained and how this is related to cilia and IFT proteins need further studies.

In summary, our data demonstrated that deletion of *IFT80* causes cilia shortened or lost. Without an intact cilium structure, activated Smo cannot move into the cilium to activate Gli, thereby inhibiting BMP2 and Runx2 signalling and OB differentiation. Meanwhile, extraciliary Smo coupled Gαi to overactivate RhoA signalling and then generate excess stress fibres, which also inhibited OB differentiation. Thus, this study reveals a new mechanism by which IFT80 plays a vital role in cilia formation and OB differentiation and is essential for the balance between canonical and non-canonical Hh pathways. In OPCs, the primary cilium provides a specialized space for accumulation of components for the canonical Hh signalling pathway. With Hh stimulation, Smo translocates to the cilium, activates Gli and subsequently drives OB differentiation by regulating BMP2 and Runx2 expression. Meanwhile, activated Smo also couples with Gαi to activate RhoA, remodelling the cytoskeleton and also providing a negative feedback to canonical Hh signalling (Fig. 7f).

Currently, there are no effective approaches to treat bone disorders caused by mutation of IFT and other ciliary proteins. Our results reveal a new mechanism regarding the role and mechanism of IFT80 in OB differentiation and bone formation, and highlight IFT80 and non-canonical Hh signalling as therapeutic targets for craniofacial and skeletal abnormalities.

## Methods

### Generation of *IFT80* conditional knockout mice

An *IFT80* gene target vector was constructed with a 5.59-kb left arm homology, a Frt-flanked Neo reporter gene and LacZ gene, a loxp-flanked exon 6 of *IFT80* and a 4-kb right arm homology. Following sequence confirmation of the construct, the targeting vector was linearized and electroporated into the C57BL/6N mouse embryonic stem (ES) cells, followed by positive selection with G418 (for Neo). Three independent ES mutant clones were confirmed by southern blot and used for generation of chimeric mice by microinjection into blastocysts of C57BL/6J mice. Chimeric mice were bred with wild-type C57BL/6J mice for generation of germ line transmission of the targeted allele. F1 mice were genotyped by PCR using tail genomic DNA samples for the presence of the target *IFT80*^*LacZNeoFlox*^ allele. Heterozygous mice were bred with *FLPeR* mice (expressing the Flpe recombinase^63^) to remove the *LacZ* and *Neo* cassette in the germ line and generate the mice with the *IFT80*^*flox*^ allele. Heterozygous mice were intercrossed and F2 progeny were genotyped for homozygous *IFT80*^*f/f*^. The homozygous *IFT80*^*f/f*^ mice were born at expected Mendelian ratio and did not exhibit any abnormalities.

To specifically inactivate *IFT80* in the OB lineage, we crossed the *IFT80*^*f/f*^ mice with *OSX-Cre* transgenic mice (also known as *OSX1-GFP::Cre*^35^), which express *Cre* under the promoter of *osterix*. In this transgenic line, Cre mainly targets the OPCs^35^.

The Cre transgene was detected using two primers CreF (5′-CCTGGAAAATGCTTCT GTCCGTTTGCC-3′) and CreB (5′-GGCGCGGCAACACCATTTTT-3′). The floxed and wild-type alleles were genotyped by two primers: IFT80S (5′-TGTGAGGCCAGCCCGA GTTA-3′); and IFT80R (5′- GCCTGAGCTACAGAGAGACCCCACG-3′), which yielded a 469 bp band for the flox allele and a 247 bp band for wild-type allele. Genotyping of mice was performed by PCR using proteinase K-digested tail DNA.

All studies and procedures performed on mice were approved by the University at Buffalo Institutional Animal Care and Use Committee.

### Primary OPCs culture and differentiation

Primary OPCs were isolated from mouse calvarial bone (4–6 pups of either sex, postnatal day 3) by serial digestion method. Briefly, calvarial bone was dissected and subjected to sequential digestions in collagenase type I (2 mg ml^−1^, EMD, Darmstadt, Germany), Trypsin (0.25%, Corning, Manassas, VA) and collagenase type I (2 mg ml^−1^) for 30 min each. Then the bone chips were subjected to collagenase type I (2 mg ml^−1^) again for 10 min. Cells from this digestion were spun down and plated in α-MEM-containing 10% foetal bovine serum (FBS), 100 U ml^−1^ penicillin and 1 μg ml^−1^ streptomycin.

OPCs from *IFT80*^*f/f*^ mice were infected with adenovirus, which overexpress either Cre (Ad-CMV-Cre, #1405, Vector Biolabs) or GFP (Ad–GFP, #1060, Vector Biolabs). Ad-CMV-Cre infection yielded 95% deletion of *IFT80* in *IFT80*^*f/f*^ OPCs, which were named as *IFT80*^*d/d*^. Ad–GFP-treated cells were used as infection control and still marked as *IFT80*^*f/f*^.

Osteogenic differentiation was induced with osteogenic medium (OS medium). OS medium is α-MEM (Gibco) containing 10% FBS, 10 mM β-glycerophosphate (Sigma, St Louis, MO), 50 μg ml^−1^ ascorbic acid (Sigma) and 10^−8^ M dexamethasone (Sigma).

ALP activity was measured at day 7 of osteogenic induction. Briefly, cells were washed with PBS and then harvested with harvest buffer containing 10 mM Tris-Cl (pH 7.4), 0.2% NP40 and 2 mM PMSF. The samples were homogenized and centrifuged. The supernatants were added to assay buffer (100 mM glycine and 1 mM MgCl_2_, pH 10.5) and *p-*nitrophenyl phosphate solution (50 mM in 0.1 M glycine buffer), and incubated at 37 °C for 15 min. NaOH solution (0.1 N) was used to stop the reaction. The optical density for ALP activity was measured at 405 nm using AD 340 microplate reader (Beckman Coulter, Fullerton, CA). ALP activity was normalized with the value of DNA content and expressed as nmol of p-nitrophenol produced per min per mg of total DNA.

To measure bone nodule formation, extracellular matrix calcium deposits were stained with Alizarin Red S solution either 14 or 21 days after osteogenic induction. Briefly, cells were fixed with 2% formaldehyde and then stained with 40 mM of Alizarin Red S solution (pH 4.4) at room temperature. The images of the stained cells were scanned. Stainings were then destained using 10% cetylpyridinium chloride in 10 mM sodium phosphate (pH 7.0) and quantified by measuring the optical density at the wavelength of 562 nm. The experiment was done in triplicate.

### Gene transfer

BMP2 and Runx2 expression adenoviruses (Ad-BMP2 and Ad-Runx2) were produced with an AdEasy system (Strategene). Full-length human *BMP2* complementary DNA (cDNA; GenBank accession no. NM_001200.1) and Runx2 cDNAs (GenBank accession no. NM_001015051.3) were inserted into AdTrack-CMV vector. The plasmids were linearized and co-transformed with pAdEasy-1 into BJ5183 cells, followed by kanamycin selection. The plasmids were confirmed by multiple restriction endonuclease analyses and then linearized and transfected into 293A cells to package adenovirus. For infection of OPCs, Ad–GFP (as a control), Ad-BMP2 or Ad-Runx2 adenovirus were added to cells in serum-free medium for 4 h, followed by incubation with 2% serum overnight^64^.

Plasmid DNA encoding pcDNA3-EGFP-RhoA-WT (Addgene plasmid #12965), pcDNA3-EGFP-RhoA-T19N (Addgene plasmid 12967, dominate negative, named as RhoA19DN) and pcDNA3-EGFP-RhoA-Q63L (Addgene plasmid 12968, constitutive active, named as RhoA63CA) were obtained from Addgene (Cambridge, MA, USA). For transfection of 5 × 10^5^ cells, 2 μg DNA with 6 μl Fugene HD transfection reagent (Promega, Madison, WI) was used. The stable transfected OPC were selected with 400 μg ml^−1^ G418 for 2 weeks.

### Reporter assay

About 1 × 10^6^ OPCs were transfected with 3 μg Gli-responsive luciferase reporter construct (8 × Gli-Luc) (gift from Dr Fernandez-Zapico^65^) and 0.6 μg pRL-TK Renilla luciferase (Promega), using Fugene HD transfection reagent. Renilla luciferase was used as internal control. Cells were induced with OS medium for 3 days, and then stimulated with 1 μg ml^−1^ of recombinant mouse Shh N-terminus (R&D systems, Minneapolis, MN) for 8 h (ref. 10). The relative luciferase activity of cell lysates was measured with the Veritas microplate luminometer (Turner Biosystem, Sunnyvale, CA) using the Dual-luciferase assay kit (Promega). The experiment was conducted in triplicate.

### RhoA activity assay

Relative RhoA activity was determined using a commercially available ELISA-based RhoA activity assay kit (G-LISA, BK#124; Cytoskeleton Inc., Denver, CO, USA) according to the manufacturer's instructions. Briefly, treated cells were lysed and snap-frozen in liquid nitrogen. Approximately 30–40 μg total protein was used for each sample. The active GTP-bound form of RhoA was detected with a specific antibody. Absorbance readings were obtained at 490 nm. Data were normalized to total RhoA assessed by total RhoA ELISA biochem kit (BK#150; Cytoskeleton Inc.). The experiment was conducted in triplicate.

### Modulators of Hh signalling and stress fibres

Purmorphamine (Tocris Bioscience, 4551) is a pharmacological activator of Smo. Purmorphamine was dissolved in DMSO and added to the cell culture at a concentration of 2 μM. Cyclopamine (Tocris Bioscience, 1623) and SANT-1 (Tocris Bioscience, 1974) were dissolved in ethanol and DMSO, respectively. The final concentration used for cells was 5 μM of cyclopamine and 100 nM of SANT-1.

Cell permeable format of C3 transferase (Cat# CT04, Cytoskeleton), covalently linked to a proprietary cell-penetrating moiety, was used to inhibit RhoA activity. PTX (Tocris Bioscience, 3097) was used to inhibit Gαi. CytoD (Sigma, C2618) and DMSO (used as control, Sigma) were used to induce transient actin cytoskeletal change. For stress fibre staining, cells were treated with 1 μM CytoD for 1 h. For cilia staining, cells were treated with 0.1 μM CytoD for 2 h following the serum starving. For osteogenesis, cells were treated with 1 μM CytoD for 1 h and, immediately after the treatment, the cells were washed twice with PBS and then incubated with OS medium. This CytoD treatment was repeated every 4 days^60^.

ROCK silencing was performed in OPCs using validated siRNA oligonucleotides that target endogenous ROCK1 (Santa Cruz, sc-36432) and ROCK2 (Santa Cruz, sc-36433). Scrambled siRNA (Santa Cruz, sc-37007) were included as controls. About 10 μmol siRNA (for 1 well in 24-well plate) were transfected by RNAimax (Invitrogen) according to the manufacturer's protocol. Efficiency of knockdown was analysed by western blot.

### Stress fibre staining and quantification

OPCs were washed twice with PBS and fixed in 3.7% formaldehyde in PBS for 10 min at room temperature. Cells were washed and permeabilized with acetone at −20 °C for 3 min. To label stress fibre, cells were blocked with 1% BSA and stained with Rhodamine–phalloidin (1:100, Invitrogen) solution for 20 min at room temperature. DAPI (6-diamidino-2-phenylindole, Invitrogen) was used as a counter stain of nuclei.

Quantifications were obtained from multichannel images using ImageJ to assess each cell image for its actin stress fibre density^66^,^67^. The F-actin images were normalized for average pixel intensity and intensity-thresholded to minimize background fluorescence. ImageJ software was used to generate line profiles. The peaks were counted and the data were expressed as number of stress fibres per cell. The experiment was conducted in triplicate.

### Immunofluorescence

To visualize cilia structure, immunofluorescence was performed using primary acetylated α-tubulin antibody (1:500, T6793, Sigma) and primary γ-tubulin antibody (1:500, T3320, Sigma). Briefly, cells were serum starved for 48 h and washed with PBS and fixed with 4% paraformaldehyde at room temperature. Fixed cells were permeabilized with 0.05% Triton X-100 and then incubated with the primary antibodies overnight at 4 °C. Alexa Fluor 568-conjugated anti-rabbit (1:1,000, A-11011, Invitrogen) or Alexa Fluor 647-conjugated anti-mouse (1:1,000, A-21235, Invitrogen) antibodies were used as secondary antibody. Counter stain of nuclei was done with DAPI. The experiment was conducted in quadruplicate.

The same procurer was used for Ki67 (Anti-Ki67, 1:300, ab16667, Abcam) staining and Smo staining. To determine Smo localization, cells were co-stained with Smo antibody (1:100, ab38686, Abcam) and acetylated α-tubulin antibody.

### MTS assay

Cells were seed at 5,000 cells per well in 96-well plate. The MTS assay was performed using the CellTiter 96 AQueous One Solution Cell Proliferation Assay kit (Promega) after 24, 48 and 72 h in culture. The optical density was measured at 490 nm.

### Whole-mount skeletal staining

Whole-mount skeletal staining with Alizarin Red and Alcian Blue was performed as previously reported with slight modification^68^. Briefly, mice were fixed in 90% ethanol for at least 1 week. Prior to staining, skin and viscera were removed. Alcian Blue solution (0.01%, in 80% ethanol and 20% glacial acetic acid) was used to stain the cartilage for 3 days. The skeletons were rehydrated in gradated ethanol to water solution. Skeletons were cleared with 1% KOH solution for 2–3 days and transferred to 1% Alizarin Red S solution (in 1% KOH) for 2–3 days to stain mineralized bone. The skeletons were then cleared with an increased gradated glycerol solution in 1% KOH, and stored in 100% glycerol for image acquisition.

### Histology and safranin O staining

Mice tibiae were excised, fixed for 24 h in 10% natural buffered formalin, and decalcified in 10% EDTA for 1–2 weeks at 4 °C. The samples were embedded in paraffin, sectioned for 5 μm and stained with H&E. Deparaffinized slides were stained with 0.1% safranin O solution for 5 min to visualize cartilage. Fast green was used as a counter stain.

### Histomorphometric analyses

Mice were i.p. injected with calcein (25 mg kg^−1^) at Day 30 and Day 34. Mice were killed 2 days after last injection and tibia were dissected, fixed, dehydrated and embedded undecalcified in a methylmethacrylate. Longitudinal sections (5 μm) were cut from the centre of the proximal tibiae with a microtome (model 2065; Reichert Jung, Heidelberg, Germany). All measurements were performed with the OsteoMeasure Analysis System (OsteoMetrics, Atlanta, GA).

### Bone micro-CT analysis

A quantitative analysis of the gross bone morphology and microarchitecture was performed with the Micro-CT system (USDA Grand Forks Human Nutrition Research Center, Grand Forks, ND). Femurs from 1-month-old *OSX;IFT80*^*f/f*^ mice and *OSX;IFT80*^*+/+*^ mice were fixed, scanned and reconstituted as three-dimensional images. Cancellous bones were evaluated in the distal femur metaphysis. About 125 slices (1.5 mm) of bone were measured to determine BV/TV (%), Tb.N (μm^−1^), Tb.Th (μm) and trabecular spacing (Tb.Sp, μm)^69^.

### Quantitative PCR

Total RNA was extracted from cultured OBs with Trizol reagent (Invitrogen, Carlsbad, CA) following the manufacturer's instructions. CDNA was synthesized from 3 μg total RNA by RNA to cDNA EcoDry Premix kit (Clontech, Palo Alto, CA). QPCR was performed on the ABI PRISM 7500 real time PCR machine (Invitrogen) with SYBR Green PCR master Mix (Invitrogen). Primers were designed with IDT SCI primer design tool (Integrated DNA Technologies, San Diego, CA). Sequence and product length for each primer pair were listed Supplementary Table 1. All reactions were run in triplicate and normalized to the housekeeping gene GAPDH. Calculations were performed according to the 2^−ΔΔCT^ method^70^.

### Western blot

Western blot was performed to detect IFT80 expression using rabbit anti-IFT80 antibody (1:400, PAB15842, Abnova). Calvarial bone from *OSX;IFT80*^*+/+*^ mice and *OSX;IFT80*^*f/f*^ mice were snap-frozen with liquid nitrogen and homogenized with RIPA buffer (50 mM Tris, 150 mM NaCl, 1% Triton X-100, 0.1% SDS, and 1% sodium deoxycholate) containing protease inhibitor cocktail (Sigma). Protein concentration was measured using BCA protein assay reagent (Pierce, Rochford, IL). Equal amounts of protein (about 20 μg) were denatured in SDS and separated in 10% SDS–PAGE gels. Proteins were transferred to polyvinylidene difluoride membranes in buffer containing 25 mM Tris, 192 mM glycine and 20% methanol. The membranes were blocked with 5% milk, incubated with primary antibody overnight at 4 °C and then incubated with horseradish peroxidase (HRP)-conjugated goat anti-rabbit IgG antibody (1:10,000, A-11034, Novex, Carlsbad, CA) at room temperature for 1 h. Visualization was performed with WesternBright ECL HRP (Advansta). GAPDH (1:2,000, A00191, Genscript) was used as the internal control.

The same procurer was used to determine the Smad1/5/8 (1:300, sc-6031-R, Santa Cruz), p-Smad1/5/8 (1:300, sc-12353, Santa Cruz), MLC2 (1:1,000, 3672, Cell Signaling technology), p-MLC2 (1:1,000, 3675, Cell Signaling technology), Cofilin (1:1,000, 5175, Cell Signaling technology), p-Cofilin (1:1,000, 3313, Cell Signaling technology), ROCK1 (1:400, sc-5560, Santa Cruz), ROCK2 (1:400, sc-1851, Santa Cruz), GFP (1:500, sc-8334, Santa Cruz) and Smo (1:1,000). The experiment was run in triplicate. Full scan results of western blots were shown in Supplementary Fig 9.

### Immunoprecipitation

Cells were treated with purmorphamine for 10 min or 4 h and then harvested with NP40 lysis buffer (150 mM NaCl, 50 mM Tris-Cl pH 7.4 and 1% NP40). Total cell lysates and 2 μg Gαi antibody (sc-136478, Santa Cruz) were incubated for 1 h at 4 °C followed by adding protein A and protein G agarose beads (Santa Cruz) with overnight immunoprecipitation. Immunoprecipitation products were washed and boiled for 10 min for western blot.

### Statistical analysis

All data are presented as mean±s.e.m. (*n*=≥3). Student's *t*-test for the comparison between two groups or two-way ANOVA followed by Tukey's multiple comparison test for grouped samples was performed. *P*<0.05 was considered to be significant. The program GraphPad Prism (GraphPad Software, Inc., San Diego, USA) was used for these analyses.

## Additional information

**How to cite this article:** Yuan, X. *et al.* Ciliary IFT80 balances canonical versus non-canonical hedgehog signalling for osteoblast differentiation. *Nat. Commun.* 7:11024 doi: 10.1038/ncomms11024 (2016).

## Supplementary Material

Supplementary Information Supplementary Figures 1-9 and Supplementary Table 1
