# Supplementary material for: Ciliary IFT80 balances canonical versus non-canonical hedgehog signalling for osteoblast differentiation
Source: Nat Commun. 2016 Mar 21;7:11024. doi: 10.1038/ncomms11024 (PMC4802171; doi:10.1038/ncomms11024)
Supplement: Supplementary Information — Supplementary Figures 1-9 and Supplementary Table 1 [file ncomms11024-s1.pdf]

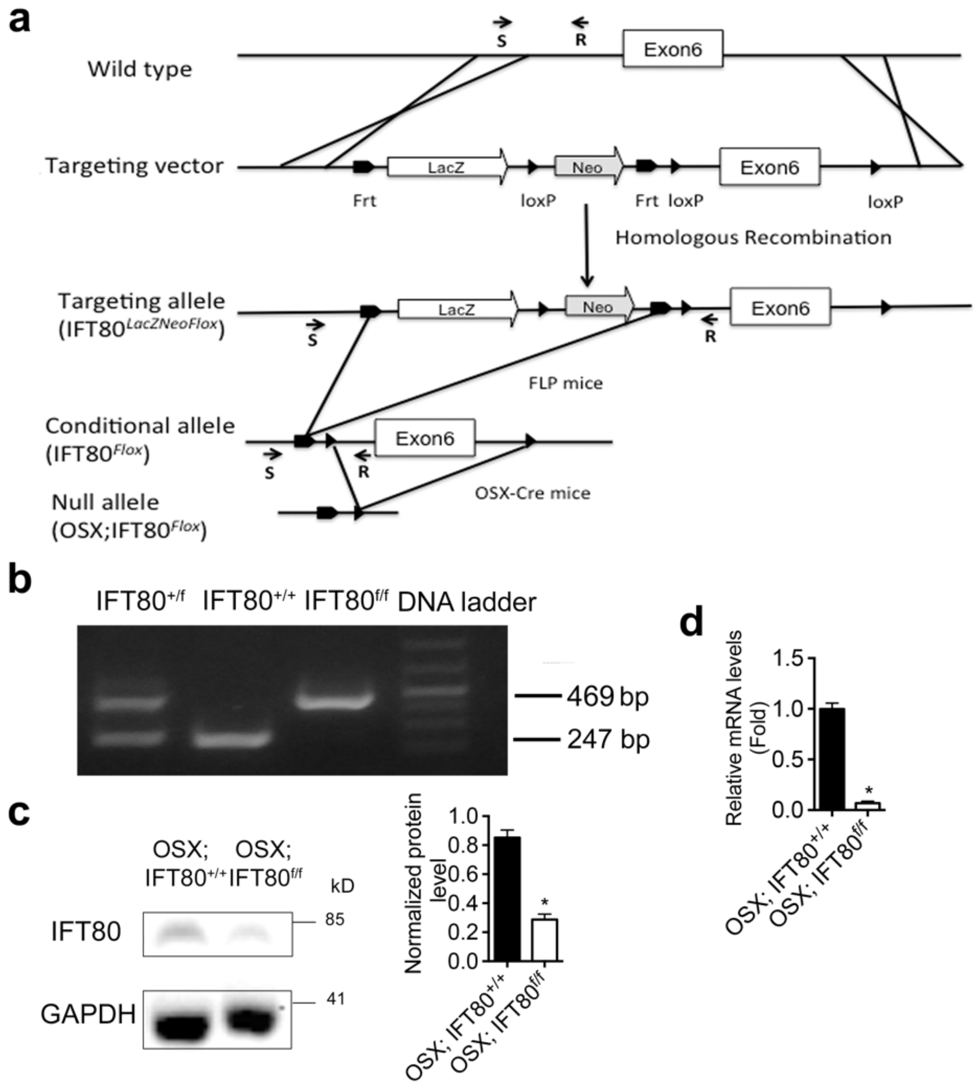

Supplementary Figure 1. OSX-Cre-mediated conditional deletion of *IFT80* from the floxed *IFT80* allele (*IFT80^{flox}*). (a) Schematic illustration of the wild-type, floxed *IFT80* allele (*IFT80^{flox}*) and *IFT80* mutant (*OSX; IFT80$^{f/f}$*). The targeting vector contains a 5.59 kb left arm homology, a Frt-flanked *Neo* reporter gene and LacZ gene, a loxp-flanked exon 6 of *IFT80*, and a 4 kb right arm homology. *IFT80^{LacZNeoFlox}* mice were mated with *FLP* transgenic mice to delete the *neo* gene and *LacZ* gene and generated *IFT80^{flox}* alleles. Deletion of the loxp cassette was achieved by Cre-mediated recombination. **(b)** Genotyping PCR analysis of *IFT80$^{f/f}$*, *IFT80$^{+/f}$* and *IFT80$^{+/+}$* alleles. The bands corresponding to the targeted mutation without *neo* gene and *LacZ* gene (*IFT80^{flox}*) is 469 bp and the bands corresponding to wild-type (*IFT80^{+})* is 247 bp. **(c)** Western blot analysis of IFT80 expression in calvarial bone from both *OSX; IFT80$^{+/+}$* mice and *OXS; IFT80$^{f/f}$* mice. IFT80 protein level was normalized to GAPDH (n = 3 mice per group). **(d)** qPCR analysis of total *IFT80* transcripts in cultured OPCs derived from *OSX; IFT80$^{+/+}$* mice and *OXS; IFT80$^{f/f}$* mice. The expression of *IFT80* is normalized to *GAPDH* expression (n = 4 mice per group). Data points indicate the means, while error bars represent s.e.m. *P<0.001 as determined by Student's *t*-test.

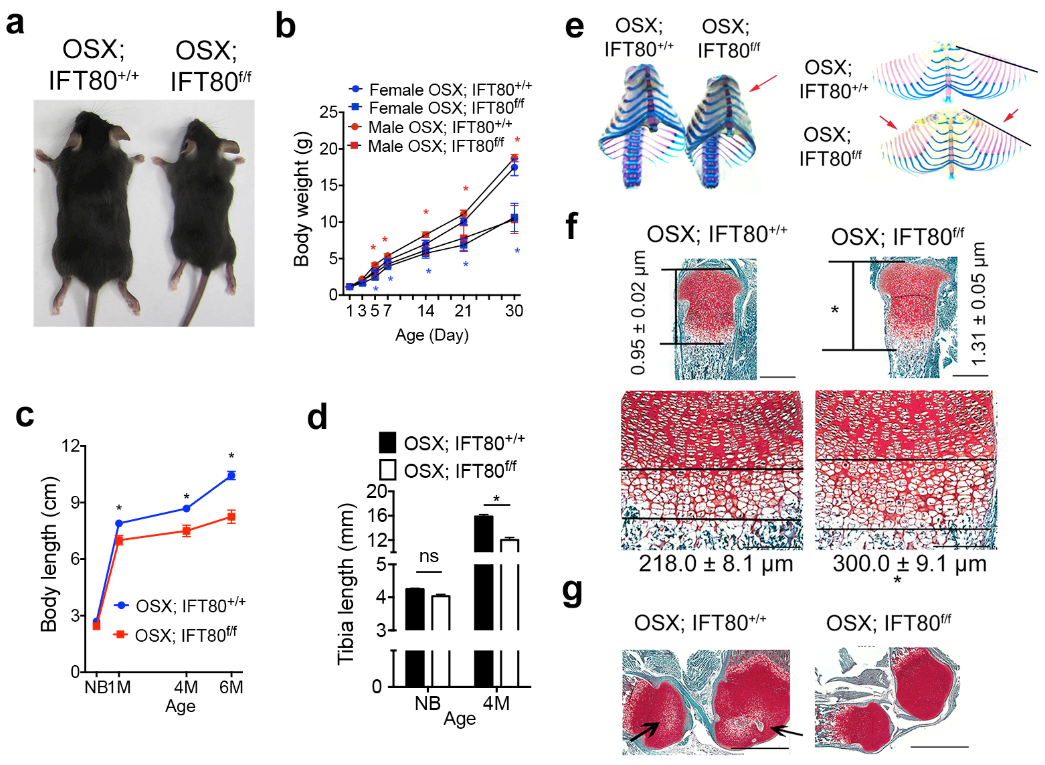

Supplementary Figure 2. Deletion of *IFT80* causes a significant growth retardation and altered cartilage development. (a) Appearance of *OSX; IFT80*$^{+/+}$ mice and *OSX; IFT80*$^{f/f}$ mice. **(b)** Body weight of *OSX; IFT80*$^{+/+}$ mice and *OSX; IFT80*$^{f/f}$ mice (n = 6 mice per group, mean ± s.e.m, *P<0.01 versus age and sex matched *OSX; IFT80*$^{+/+}$ mice, multiple *t*-test). **(c)** Body length of *OSX; IFT80*$^{+/+}$ and *OSX; IFT80*$^{f/f}$ mice at different ages. Body length was measured from nose to the base of the tail. *OSX; IFT80*$^{f/f}$ mice displayed significantly lower body length from 1 month (n = 5 mice per group, mean ± s.e.m, *P<0.01, multiple *t*-test). **(d)** Tibia length of *OSX; IFT80*$^{+/+}$ and *OSX; IFT80*$^{f/f}$ mice at newborn and 4-month old (n = 6 mice per group, mean ± s.e.m, *P<0.0001, Student's *t*-test). **(e)** The skeletons of P14 *OSX; IFT80*$^{+/+}$ and *OSX; IFT80*$^{f/f}$ mice were stained with Alizarin Red S for bone, followed by Alician blue staining for cartilage. Narrow rib cage in *OSX; IFT80*$^{f/f}$ mice were found (indicated by red arrows). **(f)** Safranin O staining of the tibia section of newborn *OSX; IFT80*$^{+/+}$ and *OSX; IFT80*$^{f/f}$ mice. *OSX;IFT80*$^{f/f}$ mice displays longer cartilage (red), especially hypertrophic cartilage (lower panel) than those in *OSX;IFT80*$^{+/+}$ mice (n = 4 mice per group, mean ± s.e.m, *P<0.001, Student's *t*-test). Scale bars represent 500 µm (top) or 200 µm (bottom). **(g)** Safranin O staining the tibia and femur section of P14 *OSX; IFT80*$^{+/+}$ and *OSX; IFT80*$^{f/f}$ mice. Arrows show the secondary ossification center formation. Scale bars represent 1 mm.

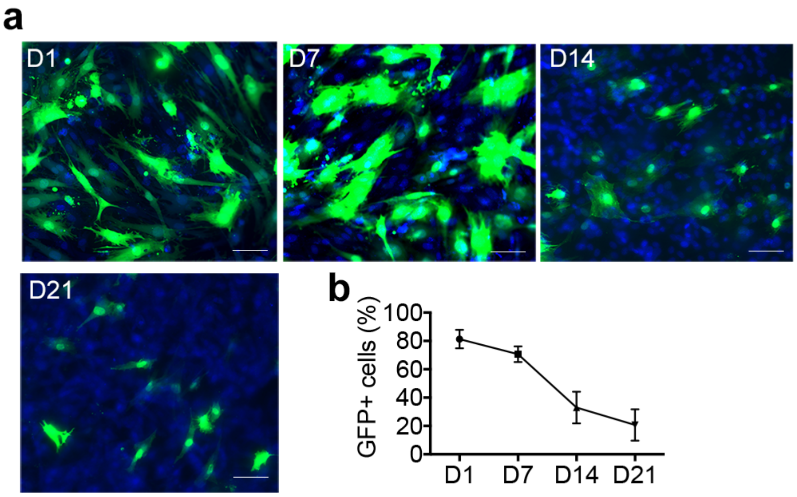

Supplementary Figure 3. GFP positive cells in Ad-GFP transfected group during OB differentiation. (a) Wild-type OPCs were transfected with Ad-GFP and then induce for osteogenesis. Cells were fixed at Day 1, 7, 14 and 21 to analyze the GFP positive cells. DAPI (nuclear marker) staining is used as counterstaining. Scale bars represent 100 μm. **(b)** Quantification of GFP positive cells population shown in a (n=3 with at least 500 cells analyzed per time point). Data points indicate the means, while error bars represent s.e.m.

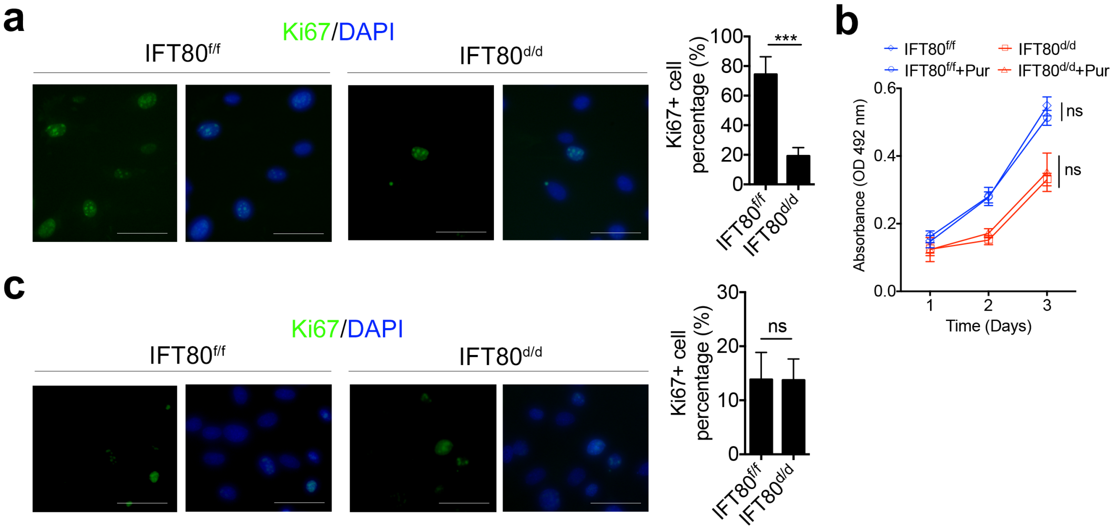

Supplementary Figure 4. Cell proliferation assay. (a) Ki67 (green) staining of *IFT80*$^{f/f}$ and *IFT80*$^{d/d}$ OPCs at the density of 500 cells per mm^2. DAPI staining is used as counterstaining to calculate the total cell numbers (n=3 with at least 200 cells analyzed). Scale bars represent 50 μm. **(b)** Proliferation (MTS) assay of *IFT80*$^{f/f}$ and *IFT80*$^{d/d}$ OPCs treated with or without purmorphamine (Pur). The starting cell density is 100 cells per mm^2 (n=3, triplicates per group). **(c)** Ki67 (green) staining of *IFT80*$^{f/f}$ and *IFT80*$^{d/d}$ OPCs at the density of 1000 cells per mm^2 (subconfluent). DAPI staining is used as counterstaining to calculate the total cell numbers (n=3 with at least 200 cells analyzed). Scale bars represent 50 μm. Data points indicate the means, while error bars represent s.e.m. ***P<0.0001 as determined by Student's *t*-test. ns: not statistically significant.

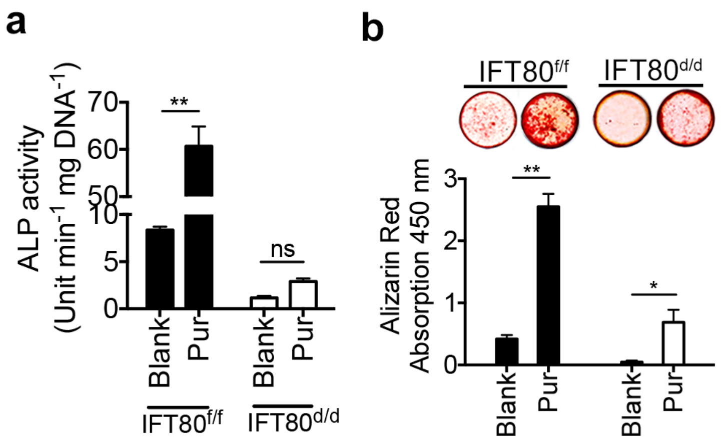

Supplementary Figure 5. Purmorphamine only partially rescues the *IFT80$^{d/d}$* OB differentiation. (a) ALP activity of *IFT80$^{f/f}$* and *IFT80$^{d/d}$* OPCs at day 7 of osteogenic induction with or without purmorphamine (Pur) stimulation (n = 4, triplicates per group). **(b)** Alizarin Red of *IFT80$^{f/f}$* and *IFT80$^{d/d}$* OPCs at day 14 of osteogenic induction with or without purmorphamine (Pur) stimulation (n = 3, triplicates per group). Data points indicate the means, while error bars represent s.e.m. Data were analyzed using Two-way ANOVA followed by Tukey multiple comparison test. *P<0.01, **P<0.0001. ns: not statistically significant.

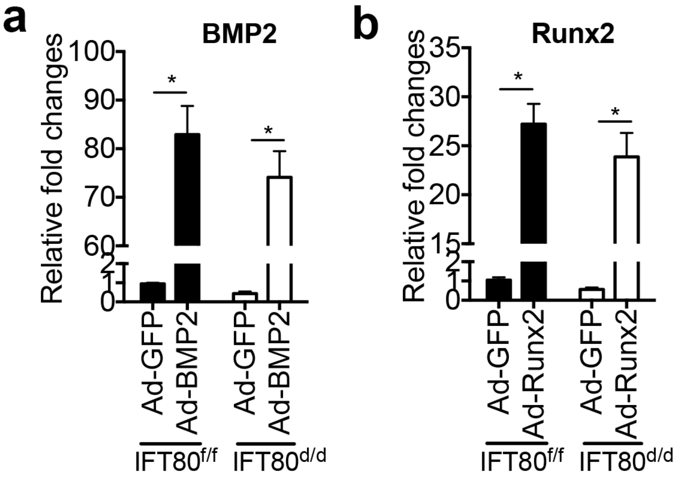

Supplementary Figure 6. Test Ad-BMP2 or Ad-Runx2 virus on *IFT80$^{f/f}$* and *IFT80$^{d/d}$* OPCs. (a) qPCR result showing *BMP2* expression in *IFT80$^{f/f}$* and *IFT80$^{d/d}$* OPCs after transfected with BMP2 adenoviruses (n = 3, triplicates per group). **(b)** qPCR result showing *RUNX2* expression in *IFT80$^{f/f}$* and *IFT80$^{d/d}$* OPCs after transfected with RUNX2 adenoviruses (n = 3, triplicates per group). Data points indicate the means, while error bars represent s.e.m. *P<0.001. Data were analyzed using Two-way ANOVA followed by Tukey multiple comparison test.

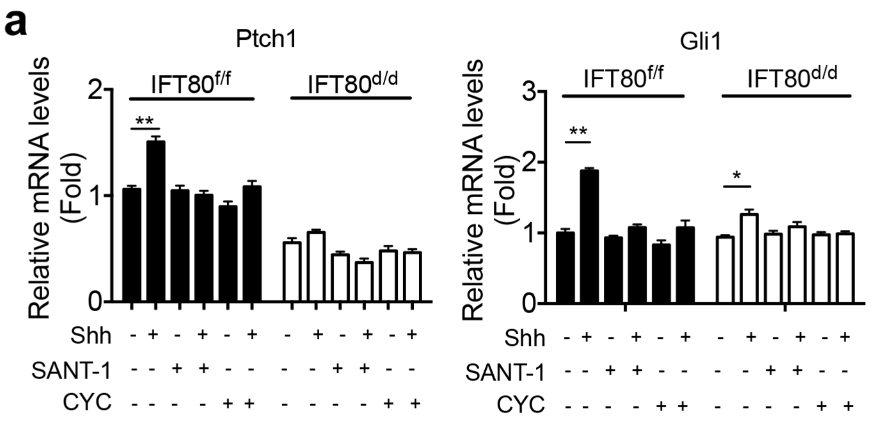

Supplementary Figure 7. SANT-1 and cyclopamine inhibits canonic Hh signaling. (a) qPCR results showing *Ptch1* and *Gli1* expression in *IFT80*$^{f/f}$ and *IFT80*$^{d/d}$ OPCs that treated with SANT-1 and cyclopamine (CYC). Data points indicate the means, while error bars represent s.e.m. Data were analyzed using Two-way ANOVA followed by Tukey multiple comparison test. *P<0.01, **P<0.0001.

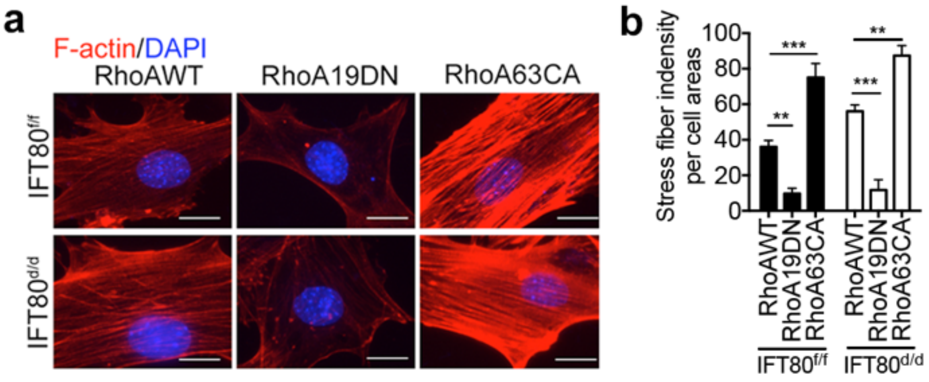

Supplementary Figure 8. Dominant negative RhoA (RhoA19DN) transfection inhibits stress fiber formation while constitutive active RhoA (RhoA63CA) promotes stress fiber formation. (a) *IFT80*$^{f/f}$ and *IFT80*$^{d/d}$ OPCs were stained to visualize stress fiber (red) and the nucleus (blue) after transfected with RhoAWT, RhoA19DN, or RhoA63CA plasmid. Scale bars represent 25 µm. **(b)** Graph quantifying the number of stress fibers shown in a (n = 3 with at least 15 cells analyzed). Data points indicate the means, while error bars represent s.e.m. Data were analyzed using Two-way ANOVA followed by Tukey multiple comparison test. **P<0.01, ***P<0.0001.

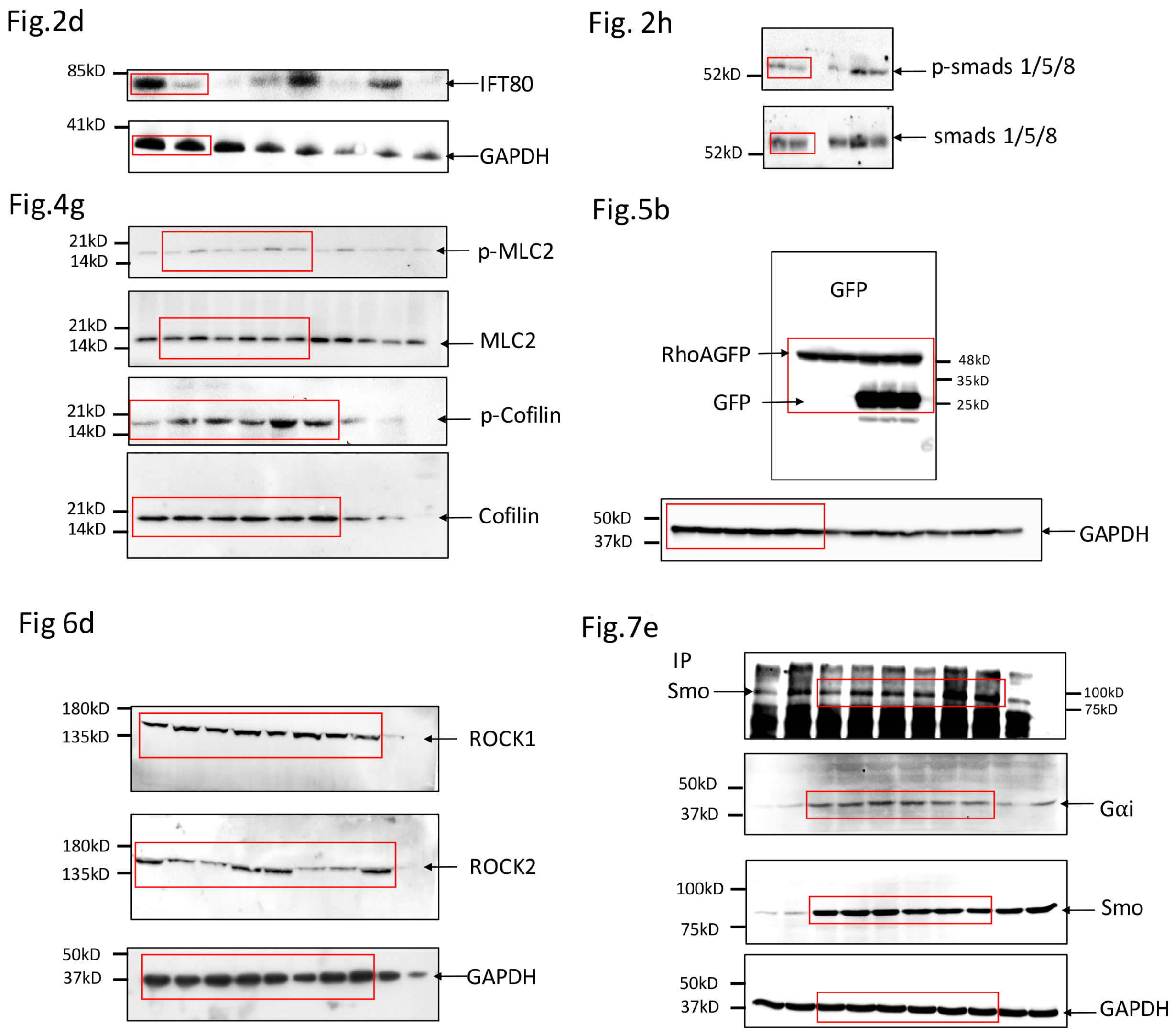

Supplementary Figure 9. Uncropped Western Blots.

Supplementary table 1. List of primers used in this study.

Gene	Forward primer sequence	Reverse primer sequence	Length
IFT80	AAGGAACCAAAGCATCAAGAATTAG	AGATGTCATCAGGCAGCTTGAC	148 bp
Runx2	ACAACCACAGAACCACAAG	TCTCGGTGGCTGGTAGTGA	106 bp
osterix	AGCGACCACTTGAGCAAACAT	GCGGCTGATTGGCTTCTTCT	121 bp
osteocalcin	AAGCAGGAGGGCAATAAGGT	ACTTGCAGGGCAGAGAGAGA	274 bp
Gli1	GGTCTCGGGGTCTCAAACTG	CCATTCTCTGGTGGGGTTCC	184 bp
Ptch1	GACCGGCCTTGCCTCAACCC	CAGGGCGTGAGCGCTGACAA	204 bp
BMP2	TCCGCTCCACAAACGAGAAA	AAAGGCATGATAGCCCGGAG	148 bp
GAPDH	TGTGTCCGTCGTGGATCTGA	TTGCTGTTGAAGTCGCAGGAG	150 bp
